# Deficiency of peripheral serotonin caused by Tph1 abrogation attenuates cerebral malaria severity

**DOI:** 10.1016/j.isci.2026.118002

**Published:** 2026-10-29

**Authors:** Antonella Turco, Gabriele Rossini, Luigi Vaccaro, Gianluca Vascelli, Martina Mandarano, Claudia Floridi, Angelo Sidoni, Giorgia Renga, Arianna Miglio, Maria Teresa Antognoni, Aurora Barbetta, Carolina Colliva, Nicola Giacchè, Marco Gargaro, Alessia Sulla, Gloria Iacomelli, Max Lombardi, Giulia Bicchieraro, Alicia Y.W. Wong, Francesca Fallarino, Vasileios Oikonomou, Teresa Zelante, Roberta Spaccapelo

**Affiliations:** 1 Department of Medicine and Surgery, Section of Genetics and Genomics, University of Perugia, Perugia, Italy; 2 Department of Chemistry, Biology and Biotechnology, University of Perugia, Perugia, Italy; 3 Department of Medicine and Surgery, Section of General Pathology and Immunology, University of Perugia, Perugia, Italy; 4 Department of Medicine and Surgery, Section of Anatomic Pathology and Histology, University of Perugia, Perugia, Italy; 5 Department of Veterinary Medicine, University of Perugia, Perugia, Italy; 6 Tes Pharma S.r.l., Solomeo, Corciano, Italy; 7 Department of Pharmaceutical Sciences, University of Perugia, Perugia, Italy; 8 Department of Medicine and Surgery, Section of Pharmacology, University of Perugia, Perugia, Italy

**Keywords:** tryptophan, cerebral malaria, *Plasmodium falciparum*, telotristat, tryptophan hydroxylase 1, Tph1, serotonin, 5-HT, *Plasmodium berghei* ANKA, Pb ANKA

## Abstract

In cerebral malaria (CM), the most severe neurological complication caused by *Plasmodium falciparum* infection, accumulating evidence suggests that tryptophan metabolism mediates disease severity by modulating inflammation and, consequently, blood-brain barrier disruption. Here, we have established an experimental CM mouse model to dissect the differential role of the host tryptophan-serotonin metabolic axis in regulating disease pathogenesis. Peripheral serotonin-deficient mice (*Tph1*^**−/−**^) displayed reduced mortality and blood-brain barrier permeability defects. In addition, we found that in the absence of serotonin synthesis via the enzyme Tph1, mice develop inflammation characterized by a mixed type 1 and type 2 cytokine signature. Pharmacological depletion of peripheral serotonin through injection of the Tph1 inhibitor telotristat in wild-type mice significantly improved resistance against CM. Thus, we propose that targeting serotonin synthesis through Tph1 antagonism could have a therapeutic role in *P. falciparum* infection and may act as a potential treatment strategy for CM.

**Video abstract:**

## Highlights

• Peripheral serotonin-deficient mice extended survival in experimental cerebral malaria
• The peripheral deficiency of serotonin preserves the integrity of the blood-brain barrier
• Telotristat protected mice from experimental cerebral malaria

## Graphical abstract

**Figure:**
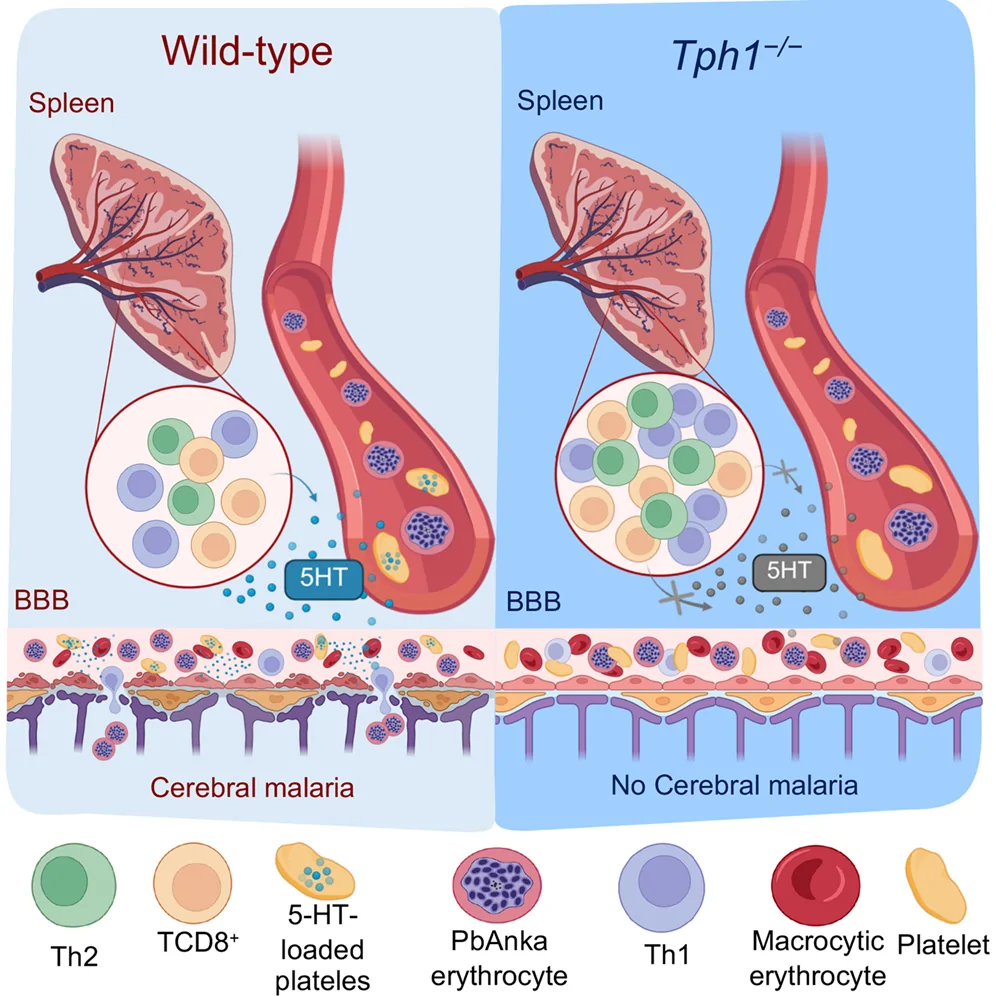

## Teaser

Neuroscience; Immunology; Parasitology

## Introduction

Serotonin [5-hydroxytryptamine (5-HT)] is a monoamine compound best known for its role as a neurotransmitter in the central nervous system, regulating sensations, behavior, and many other cerebral functions. It is mainly produced by two different enzymes, which are “rate-limiting” for 5-HT synthesis: tryptophan hydroxylase (Tph)1 and Tph2.^1^^,^^2^ Tph1 mediates 5-HT production in peripheral tissues,^3^ while Tph2 is expressed in the central nervous system. Interestingly, the two compartments are independent in homeostasis, as the amount of 5-HT in the brain is not affected by peripheral concentrations. The majority of 5-HT released is found in platelets and stored in dense granules via the 5-HT transporter (SERT/SLC6A4).^4^ One of the mechanisms recently demonstrated is that mast cells play a pivotal role in regulating platelet activation and, consequently, their degranulation.^2^^,^^5^ 5-HT is also able to covalently bind glutamine and G-proteins and determine the so-called post-translational modification “serotonylation,”^6^ which triggers a-granule exocytosis from platelets and eventually thrombosis.^6^ In addition, platelet-derived 5-HT modulates steady-state leukocyte rolling mediated by P-selectin. In the absence of platelet 5-HT, the reduced neutrophil interaction with the vessel wall results in defective neutrophil recruitment to sites of acute inflammation.^7^

Malaria is an infectious disease caused by a protozoan parasite of the *Plasmodium* genus. It is transmitted by *Anopheles* mosquitoes. Malaria is now the most prevalent parasitic disease in low-income countries, with 282 million new cases annually (94% of which occurs in the African Region).^8^ Over the past 20 years, international agencies, local governments, and private foundations have made a coordinated effort to reduce the incidence. This has led to a reduction of more than 40% worldwide. However, these improvements have stalled in recent years, with malaria still killing more than 600,000 people each year, two-thirds of whom are children under the age of 5. Children in sub-Saharan Africa are particularly vulnerable to cerebral malaria (CM), the most serious neurological complication of the disease caused by *Plasmodium falciparum* infection. Mortality as a result of CM ranges from 15% to 20% and leaves up to 25% of survivors with long-term neurological sequelae. Although available antimalarial drugs are effective in eliminating parasites from the blood, they have no specific effects against CM.^9^ It is almost well established that in severe cases, peripheral 5-HT serum levels are extremely low, while Indoleamine 2,3-dioxygenase (IDO)-derived metabolites, such as kynurenines, increase. Very recently, it was shown that the reduced amount of blood 5-HT also affects mosquitoes’ ability to transmit *Plasmodium*.^10^ Certain antimalarial drugs have been found to modulate 5-HT levels in the synapses and platelets. While some of the antimalarial-mediated increase in neuronal 5-HT levels can be attributed to the fact that they activate K^+^ channels, other antimalarial agents bind to SERT and inhibit 5-HT reuptake.^11^

*Tph1*^−/−^ mice contain normal levels of 5-HT in the brain but are depleted of the monoamine in the peripheral tissues and fluids. It was recently shown that *Tph1*^−/−^ mice suffer from macrocytic anemia, decreased red blood cell survival, and increased sensitivity to red blood cell phagocytosis.^12^ *Tph1*^−/−^ mice show greater resistance in a mouse model of LPS-induced septic shock, indicating higher immune reactivity.^13^ More recently, it has been shown that CD8^+^ T cells express Tph1 and affect antitumor responses.^14^ In this context, antitumor CD8^+^ T cells are affected by serotonylation, which reduces their role in pancreatic cancer.^15^

Like other parasitic infections, malaria can trigger a Th2-type immune response characterized by allergic inflammation, including activation of mast cells and the release of histamine.^16^^,^^17^^,^^18^

An important aspect, still not fully investigated, is that allergic inflammation has been known to reduce 5-HT release via histamine receptors. Thus, in severe malaria, a high blood level of histamine is observed, but a low amount of 5-HT is measured.^10^ This effect may also be related to the increase of basophils during malaria infection that is leading to histamine release. Recently, the increased level of basophils was also observed in concomitance with a predominant role of B cells in CM.^18^^,^^19^

In general, a cytotoxic Th1 immune response balanced with an antibody-mediated Th2 immune response is required to clear parasites while preventing immunopathology as a result of an overwhelming Th1 response. However, a Th2-biased immune response is less effective than a Th1 response for clearing intracellular parasites.

Here, we have used a mouse model of experimental CM (ECM) to compare the susceptibility of wild-type (WT) mice to mice deficient in peripheral 5-HT. The results clearly show that genetic deficiency or pharmacological inhibition of peripheral 5-HT biosynthesis enhances resistance to *Plasmodium*.

In terms of inflammatory response, Tph1 abrogation leads to an increased peripheral inflammation related to Th1 response and increased CD8^+^T cells in cervical lymph nodes not associated to higher amount of plasma IFN-γ. Interestingly, a balanced type 2 cytokine response is highlighted in Tph1-deficient mice. The absence of Tph1 had also a clear impact on vascular permeability since *Tph1*^−/−^ mice brain resulted in negative upon Evans Blue inoculation in comparison with WT mice.

Thus, although peripheral 5-HT is lower than other inflammatory mediators in CM, results show that it is playing a major role in the pathogenesis of the disease, since the abrogation of the Tph1 axis in the periphery completely changes the whole pathogenic scenario.

## Results

### Peripheral 5-HT-deficient mice are protected during experimental CM

We have recently demonstrated that Tph1 is highly expressed in mast cells, particularly within peripheral lymph nodes, in experimental autoimmune encephalomyelitis (EAE), a mouse model of multiple sclerosis.^2^ Consistent with this observation, multiple lines of evidence indicate that Tph1 mediates immune-tolerogenic responses in EAE.^20^ Therefore, peripheral serotonin-deficient (*Tph1*^−/−^) mice develop increased encephalitogenic T cell expansion and higher CD8^+^ T cell activation, compared with their WT counterparts.^2^^,^^21^

Here, we have exploited both *cKit*^−/−^ and *Tph1*^−/−^ mice, which lack mast cells and peripheral 5-HT production, respectively, to model key aspects of human CM.

Mice underwent an ECM, in which they were injected intraperitoneally with *Plasmodium berghei* ANKA (*Pb* ANKA)-infected red blood cells (iRBCs) (Figure 1A). Compared with WT controls, both *cKit*^−/−^ and *Tph1*^−/−^ mice showed increased survival (Figure 1B). Notably, *Tph1*^−/−^ mice did not develop ECM, like the other groups, even if the parasitemia was noted to grow faster (Figure 1C). To better mimic the natural life cycle of the parasite, another ECM was performed by injecting mice with *P. berghei* sporozoites intravenously (Figure 1D). Both *cKit*^−/−^ and *Tph1*^−/−^ mice showed again increased survival, with *Tph1*^−/−^mice not developing ECM and surviving beyond three weeks post-infection, and a similar trend in terms of parasitemia (Figures 1E and 1F).

**Figure 1:**
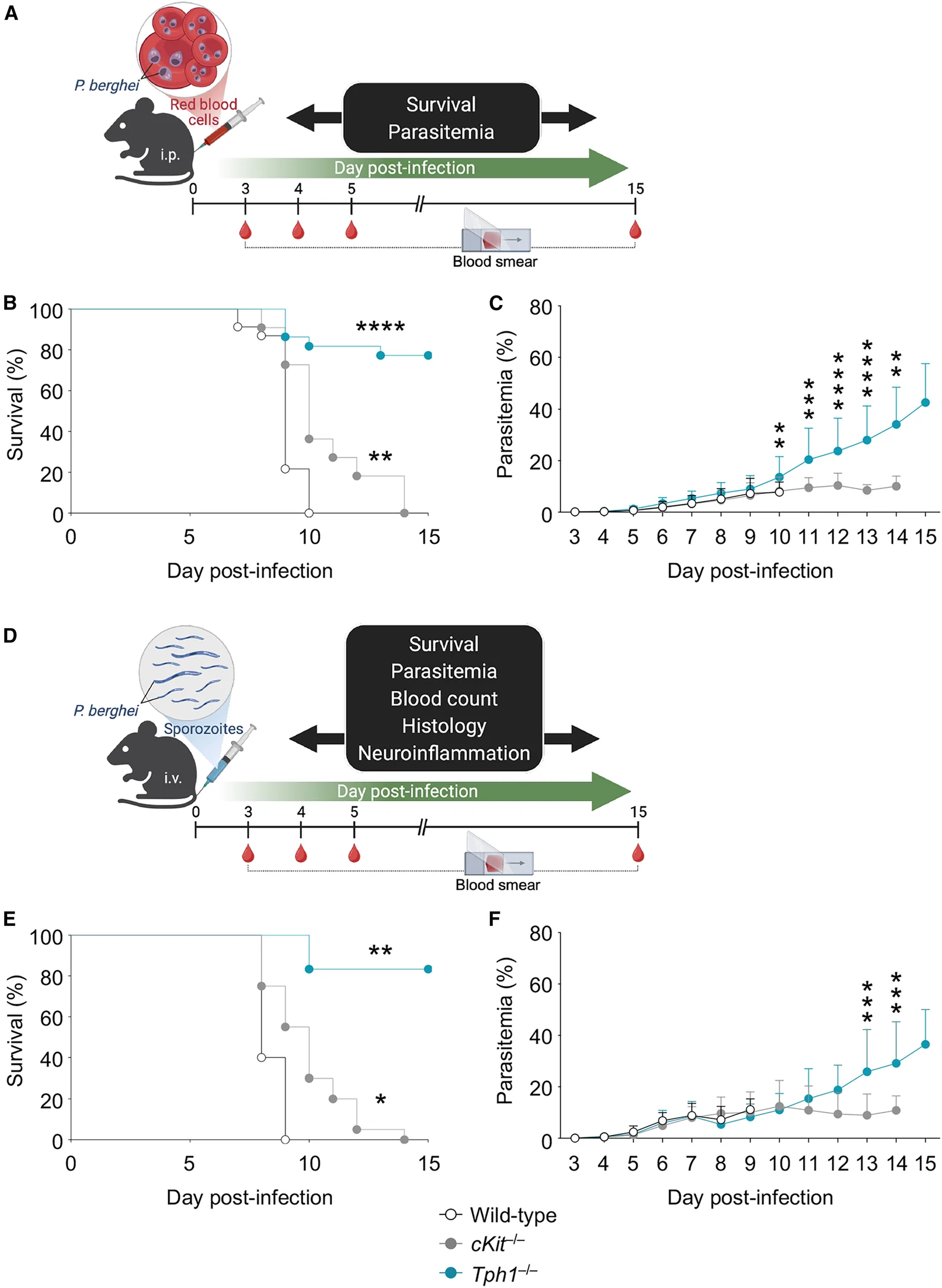
Peripheral 5-HT-deficient mice show prolonged survival to *Plasmodium berghei* ANKA infection (A–C) Experimental schematic (A), survival (B), and parasitemia levels (C) of wild-type, *cKit*^−/−^, and *Tph1*^−/−^ mice injected intraperitoneally (i.p.) with *Pb*A-infected red blood cells (*n* = 10 per group; four experiments). (D–F) Experimental schematic (D), survival (E), and parasitemia levels (F) of wild-type, *cKit*^−/−^, and *Tph1*^−/−^ mice injected intravenously (i.v.) with *P. berghei* sporozoites (*n* = 10 per group; four experiments). All quantitative data represent mean ± SEM. ∗*p* < 0.05, ∗∗*p* < 0.01, ∗∗∗*p* < 0.001, ∗∗∗∗*p* < 0.0001 as calculated using log rank (Mantel-Cox) test (B and E), unpaired *t* test (F), days 11 and 12, unpaired *t* test with Welch’s correction [(C), days 11, 12 and 14, and (F), days 13 and 14], or Mann-Whitney *U* test (C), days 10 and 13.

To assess the development of ECM, the animals were monitored daily, and the following physical and behavioral aspects were evaluated: ruffled fur, unsteady gait, partial paralysis, seizures, and coma. *Tph1*^−/−^ mice showed no visible signs of illness throughout the observation period compared with both *cKit*^−/−^ and control mice, which instead showed progressive deterioration from days 6–10 post-infection. These observations further confirmed that mice lacking the Tph1 enzyme do not exhibit the typical clinical picture of mice with ECM (Figure S1).

### The integrity of the blood-brain barrier is preserved in Tph1^−/−^ mice

Histopathological analysis of the central nervous system shows that *Tph1*^−/−^ mice infected with *P. berghei* sporozoites intravenously have fewer signs of hemorrhages in both the hypothalamus and the olfactory bulb (Figures 2A and 2B) compared with *cKit*^−/−^ and control mice. Also, as expected, the number of thrombi is significantly reduced in *Tph1*^−/−^ mice in the central nervous system, as already shown.^6^ The reason why the histopathology is altered in ECM is due to loss of integrity of the blood-brain barrier (BBB), which is a pathological feature of lethal neuroinflammation in ECM-susceptible mice that can be assessed by Evans blue extravasation (Figures 2C and 2D).

**Figure 2:**
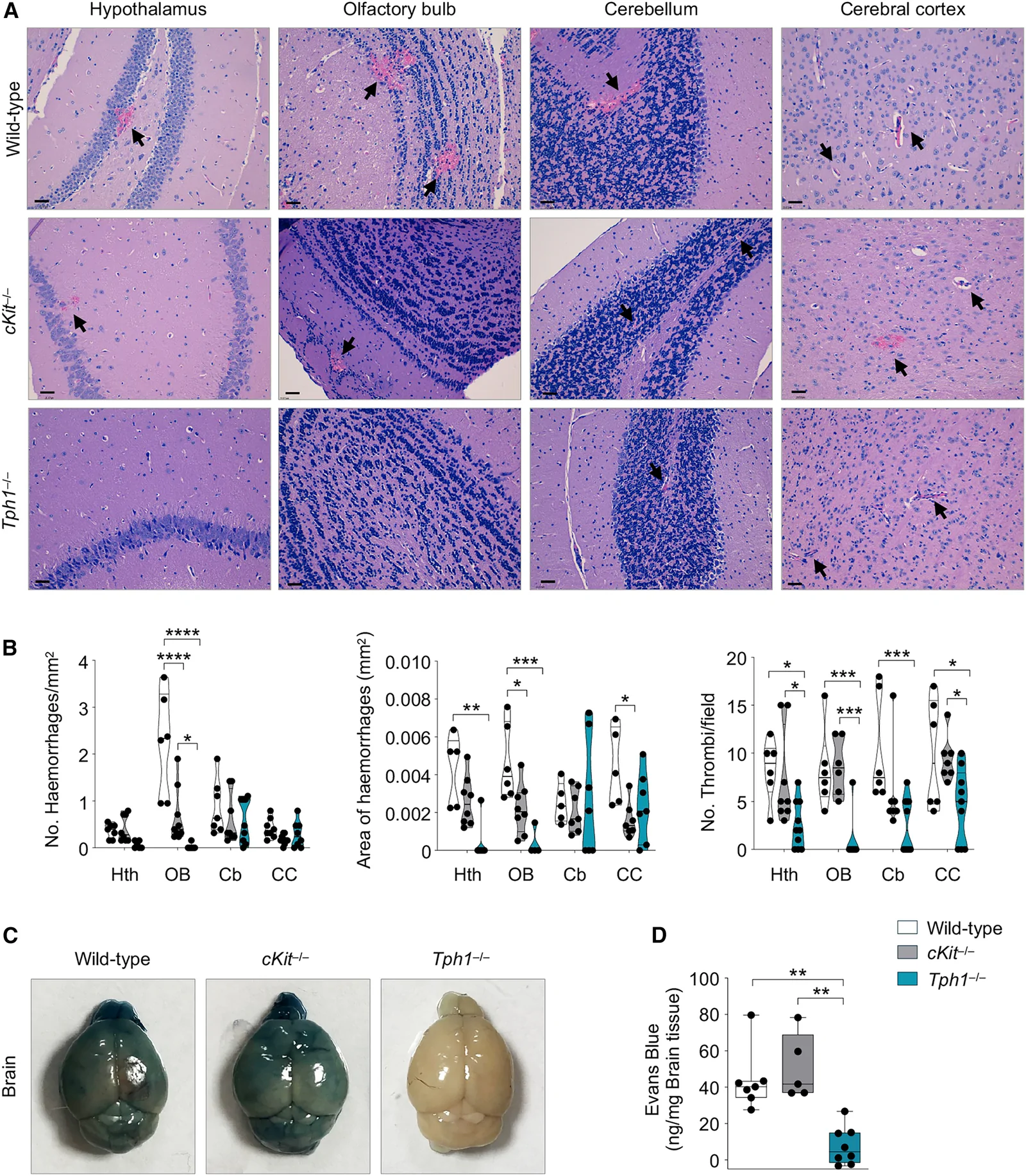
5-HT peripheral deficiency preserved blood-brain barrier integrity in a mouse model of cerebral malaria (A and B) Representative H&E-stained hypothalamus (Hth) (left), olfactory bulb (OB) (middle left), cerebellum (Cb) (middle right) and cerebral cortex (CC) (right) tissues (A) and pathology score (B) from wild-type, *cKit*^−/−^, and *Tph1*^−/−^ mice following intravenous injection with *Pb*A-sporozoites (*n* = 6–10 per group; three experiments). (C and D) Representative macroscopic view of dye-stained brains (C) and Evans Blue quantification (D) (*n* = 5–8 per group; two experiments) from wild-type, *cKit*^−/−^, and *Tph1*^−/−^ mice infected as described above. Images were captured at 200× magnification. Scale bars, 20 mm. Each dot represents an individual mouse. All quantitative data represent mean ± SEM. ∗*p* < 0.05, ∗∗*p* < 0.01, ∗∗∗*p* < 0.001, ∗∗∗∗*p* < 0.0001 as calculated using two-way ANOVA with Turkey’s multiple-comparisons test (B), or Kruskal-Wallis *H* test with Dunn’s multiple-comparisons test (D). The arrows in (A) highlight the pathological parameters analyzed in (B).

The integrity of the BBB represents another important indicator for assessing the development of ECM. The animals were inoculated with Evans Blue solution, which binds to plasma albumin and can stain the brains of mice in the presence of a BBB permeability defect. The results showed that the brains of control mice and *cKit*^−/−^ mice that died from CM were stained blue, whereas the brains of *Tph1*^−/−^ mice showed no staining, indicating an intact BBB. Quantitative analysis of the penetrated dye also confirmed what was observed in the images, with the *Tph1*^−/−^ group values being significantly lower than the control (Figure 2D). The results suggest that *Tph1*^−/−^ mice have lower BBB permeability compared with *cKit*^−/−^ and control mice, following intravenous infection with *P. berghei* sporozoites. In addition, we have also monitored the murine coma and behavior scale for quantitative assessment of murine CM and to understand how parasitemia was impacting the overall clinical status of *Tph1*^−/−^mice. *Tph1*^−/−^mice showed no neurological signs of CM compared with control mice (Figure S1).

Then, we assessed the effect of the complete deficiency of Tph1 on hematological parameters at 7- and 15-day post-infection (Figure 3). After 7 days post-infection, *Tph1*^−/−^mice survived the disease, but at 15 days exhibited a significant decrease in RBC count, hematocrit, and hemoglobin total concentration, mean corpuscular hemoglobin (MCH) content, and concentration (MCHC) (Figures 3A–3E). Mice that survived the acute phase developed severe anemia, and platelet (PLT) count per microliter was slightly reduced (Figure 3F). Notably, they also developed significant RBC macrocytosis (MCV) at 15 days post-infection, as expected (Figure 3G).^22^ In addition, we measured total white blood cell (WBC) counts and found them to be lower in *Tph1*^−/−^mice (Figure 3H) at 7 days compared with control mice, while at 15 days, the amount of WBC is significantly higher in mice with Tph1 deficiency that survived infection. Therefore, results show that Tph1 deficiency is associated with lower BBB permeability compared with controls. In addition, following survival to the intravenous infection, *Tph1*^−/−^mice do not control blood parasitemia and therefore develop strong anemia and leukocytosis at 15 days post-infection.

**Figure 3:**
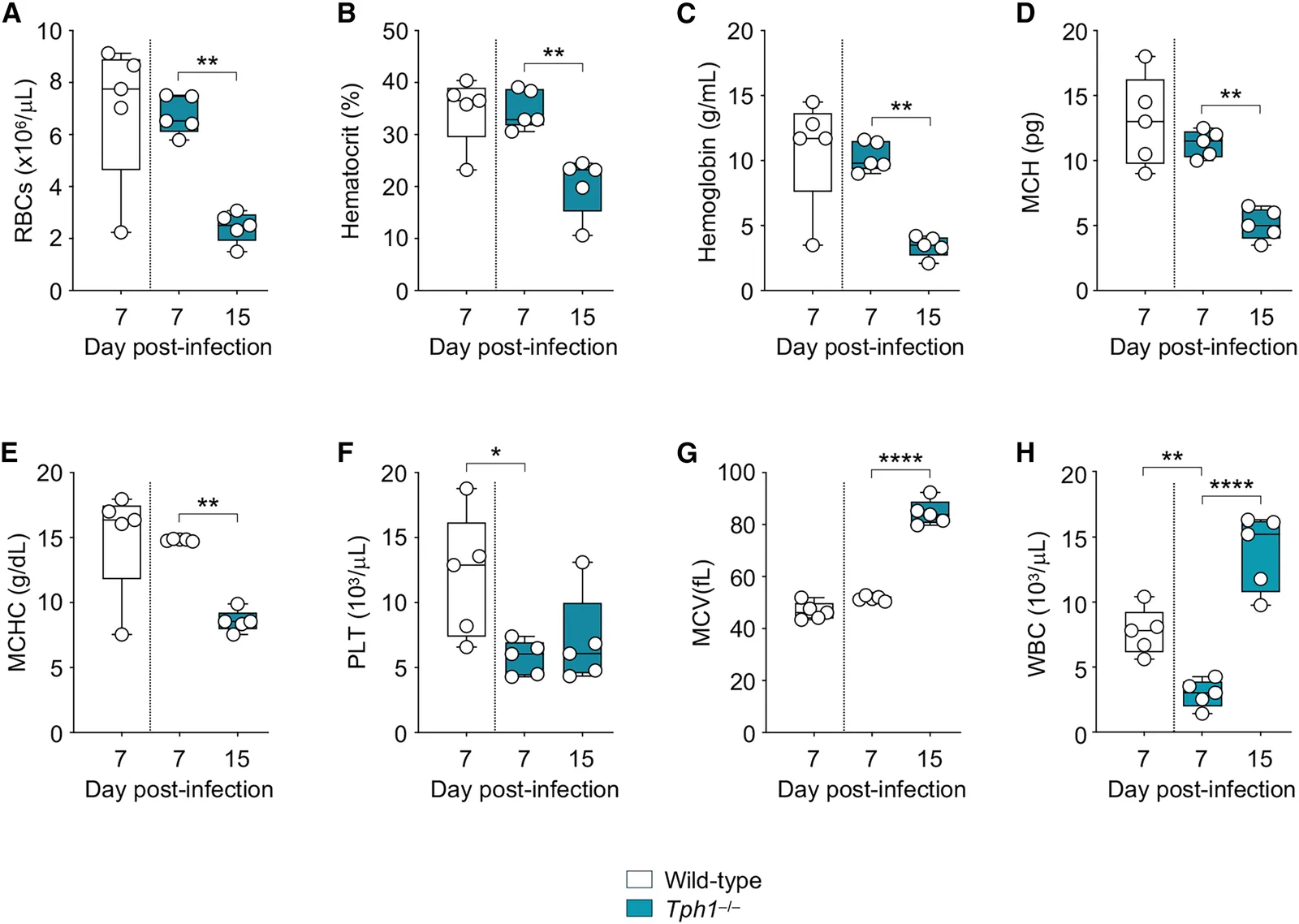
*Tph1*^−/−^ mice develop systemic inflammation during cerebral malaria (A–G) Red blood cell (RBC) count (A), hematocrit (B), and hemoglobin (C) concentration, mean corpuscular hemoglobin (CMH) (D), mean corpuscular hemoglobin concentration (MCHC) (E), platelets (PLT) (F), mean corpuscular volume (MCV) (G), and whole blood count (WBC) (H) from wild-type and *Tph1*^−/−^ mice injected with *Plasmodium berghei* sporozoites intravenously at 7 and 15 days post-infection (*n* = 5 per group; two experiments). Each dot represents an individual mouse. All quantitative data represent mean ± SEM. ∗*p* < 0.05, ∗∗*p* < 0.01, ∗∗∗∗*p* < 0.0001 as calculated using one-way ANOVA with Bonferroni’s multiple comparisons test (A–G).

### Tph1 deficiency leads to a systemic inflammatory response dominated by a mixed type 1 and type 2 signature

Since the resistance by using the ECM was induced in *Tph1*^−/−^ mice, we decided to investigate their peripheral immune profile, also knowing that peripheral, non-neuronal 5-HT released from aggregating platelets at sites of vascular injury is a known mitogen for vascular endothelial cells as well as a molecule able to recruit immune cells.^7^ Since *Tph1*^−/−^ mice displayed higher resistance compared with mice deficient in mast cells (*cKit*^**−/−**^*)* against PbANKA*,* we sought to understand if Tph1 might be expressed in additional cellular compartments, particularly those involved in the pathogenic process of CM. We found that in mice injected with *P. berghei* sporozoites intravenously, Tph1 is expressed in the spleen (Figures S2A and S2B) and in CD8^+^ T cells isolated from the spleen in ECM, as already found in the cancer microenvironment (Figure S2C).^14^^,^^15^ *Tph1*^−/−^mice were sacrificed and plasma collected for a Luminex-based detection assay. Thus, we analyzed 48 parameters in the murine plasma simultaneously to understand the immune profile in both *Tph1*^−/−^ and control mice (Figure S5).

We found that at 10 days post-infection, *Tph1*^−/−^ mice were expressing higher levels of the cytokine BAFF (Figure 4A), which may underline a B cell-dependent response and justify the hyperplasia of germinal centers in cervical lymph nodes (Figures S3A and S3B).

**Figure 4:**
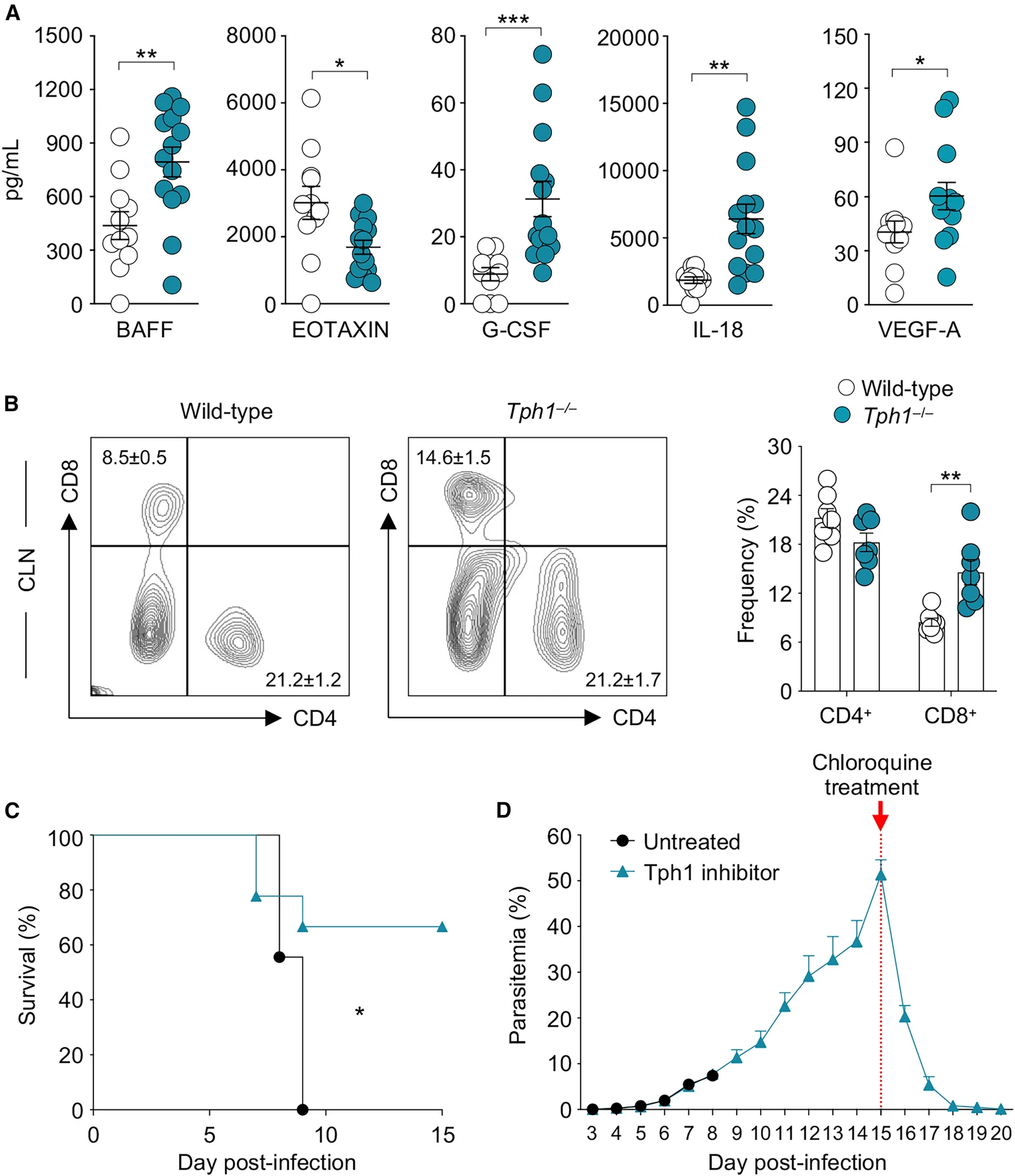
Pharmacological depletion of Tph1 activity and chloroquine treatment synergize to enhance BBB integrity, protecting mice in a cerebral malaria model (A) BAFF, EOTAXIN, G-CSF, IL-18, and VEGF-A levels from wild-type and *Tph1*^−/−^ mice infected intravenously with *Plasmodium berghei* sporozoites (*n* = 2–7 per group; three experiments). (B) Representative contour plots (left) and frequency (right) of CD4^+^ and CD8^+^ T cells in wild-type and *Tph1*^−/−^ CLN tissue (*n* = 7 per group; three experiments) in mice infected as in (A). (C and D) Survival (C) and parasitemia levels (D) in wild-type mice infected with *PbA* sporozoites and treated intraperitoneally once daily for 3 consecutive days, starting on day 3 post-infection with either vehicle or telotristat ethyl (*n* = 9 per group; 50 mg/kg; three experiments). Each dot represents an individual mouse. All quantitative data represent mean ± SEM. ∗*p* < 0.05, ∗∗*p* < 0.01, ∗∗∗*p* < 0.001 as calculated using unpaired *t* test with Welch’s correction (A), BAFF, EOTAXIN, G-CSF, and IL-18, Mann-Whitney *U* test (A), VEGF-A, or two-way ANOVA with Bonferroni’s multiple-comparisons test (B), frequency of CD8^+^ T cells. ∗*p* < 0.05 as calculated using log rank (Mantel-Cox) test (C). See Figures S1–S5.

The chemokine EOTAXIN is significantly reduced, which is a sign of a reduced type 2 immunity compared to WT mice, while G-CSF and IL-18 are increased, underlining a type 1 immunity up-regulated in the condition of 5-HT reduction (Figure 4A). The growth factor for endothelial cells (VEGF-A) is also increased in *Tph1*^−/−^ mice, which is the result of high inflammation but also endothelial repair (Figure 4A). Thus, although *Tph1*^−/−^ mice are relatively highly activated in terms of type 1 immunity, the absence of 5-HT is pivotal in controlling the endothelial damage and therefore the severity of ECM.

Overall, the inflammatory scenario observed in *Tph1*^−/−^mice as shown by the Luminex assay (Figure S5), appears characterized by type 1 cytokines but also some type 2 cytokines, such as IL-13 and IL-5. Interestingly, the anti-inflammatory cytokine IL-10 is upregulated in the absence of Tph1, which may underline a substantial immunoregulatory network upon infection. The mixed type 1 and type 2 signatures may identify a condition where the immune response will be preventing excessive inflammation and tissue damage, as shown in the literature.^23^

In terms of cellular recruitment, *Tph1*^−/−^mice as shown by FACS analysis, show an increased number of CD8^+^ T cells compared with control WT mice in peripheral cervical lymph nodes (Figure 4B), although we can’t assume the changes in the absolute number of cells in the cervical lymph nodes (see limitations of the study section).

To investigate the levels of 5-HT during infection, we have analyzed the plasma levels of a targeted panel of tryptophan-derived metabolites in *Tph1*^−/−^ and control mice infected with PbANKA sporozoites intravenously. We have found extremely low levels of peripheral 5-HT during infection in both strains of mice, considering that naive WT mice plasma 5-HT showed a mean of 3,337.46 nM (SD = 498.50).

The low amount of 5-HT in WT-infected mice may be related to several mechanisms as diminished uptake of tryptophan in the gastrointestinal tract, reduced storage in platelets due to thrombocytopenia, and increased turnover by serotonin-metabolizing enzymes (Figures S4A and S4B). The low amount of 5-HT in *Tph1*^−/−^ mice was expected, since the 5-HT biosynthesis is genetically deficient, while the production of kynurenic acid (KYNA) is relatively high. The levels of tryptophan are similar in the two strains of mice, underlining the ability of *Tph1*^−/−^ mice to degrade tryptophan through alternative pathways. Interestingly, the metabolism of tryptophan via the mouse microbiota is shown by the increased amount of indole-3-aldehyde (I3A) and indole-3-propionic acid (IPA). These latest findings underscore the ability of *Tph1*^−/−^ mice to uptake tryptophan in the gut and switch to tryptophan degradation into microbial metabolites with known immunomodulatory effects (Figure S4B).

Therefore, the genetic deficiency of Tph1 leads to a major control of the systemic inflammatory response against the parasite, and a slight increase in plasma microbial indole-derivative metabolites.

### Pharmacological depletion of TPH1 activity enhances the resistance of mice to CM

To further investigate the results obtained in *Tph1*^−/−^ mice, WT mice infected intravenously with *Pb* ANKA sporozoites were treated with telotristat etiprate, whose therapeutic target is the Tph1 enzyme. A class of xanthine-imidazopyridine and -imidazothiazole Tph inhibitors to reduce pathologically high serotonin synthesis in the periphery has been produced recently. The peripheral inhibition ameliorates pulmonary hypertension in a rat model of the disease.^24^^,^^25^ We have used the drug telotristat, which is able to block selectively Tph1 and not Tph2 because it does not pass the BBB. The Tph1 inhibitor, telotristat, has been released on the market for the treatment of carcinoid syndrome diarrhea.^26^

The animals were intravenously infected with *P. berghei* sporozoites and subsequently treated intraperitoneally with 50 mg/kg of telotristat etiprate on days 3, 4, and 6. As a control, a second group of animals was treated with the vehicle used to dissolve the drug. Mice treated with the drug showed a 67% survival rate, which was significant compared with control mice, all of which died between the 8th and 9th days post-infection (time window of ECM) (Figure 4C). Pharmacological treatment did not alter parasitemia compared with control mice in the early phase of the infection, as previously observed in *Tph1*^−/−^ mice (Figure 4D). The drug does not have a direct effect on the parasite, as clearly shown in the treated animals, which subsequently showed an increase in parasitemia, reaching about 50% around day 15 post-infection.

Mice that survived the infection and had previously been treated with telotristat were then administered with chloroquine, a drug used in malaria treatment with a direct effect on the parasite, daily for six days. As it is known, treatment with chloroquine eliminates all parasites present in the blood, bringing parasitemia levels to 0%. The results obtained confirmed the pattern, in terms of survival and parasitemia levels, previously observed following infection with PbANKA in *Tph1*^−/−^ mice. Thus, we wanted to mimic this condition by reducing Tph1 activity *in vivo* in WT mice. The treatment couldn’t control the overall parasitemia; however, by subsequently treating mice with cloroquine, it was possible to ameliorate both mouse survival and parasite load (Figures 4C and 4D).

## Discussion

Data have highlighted how the absence of Tph1 activity in controlling 5-HT levels in the periphery can be exploited for treating CM.

In malaria, several pathogenic roles have been identified for inflammatory mediators, such as interleukins or chemokines, in damaging the BBB and allowing cerebral invasion by the parasite. Also, metabolic products associated with immune regulation play a central role in CM pathogenesis. More recently, it has been found that parasites weaken the human endogenous tryptophan metabolism and further affect *Plasmodium* transmission, therefore affecting the mosquitoes’ overall fitness. Indeed, it has been described in CM that a precise metabolic signature is associated with severe pathology.^10^ It is well accepted that a low level of plasma 5-HT arising from tryptophan metabolism is a sign of severe CM. The low levels of peripheral 5-HT during CM are mainly due to platelet activation and degranulation, and its rapid degradation. Conversely, a high level of tryptophan-derived kynurenine is observed following IDO activation. Although IDO-1 is not directly involved in the pathogenesis of CM in C57BL/6 mice, the production of the kynurenine pathway metabolite, such as picolinic acid, may contribute to the development of murine CM.^27^ Histamine levels are also elevated in severe disease, mainly due to expanded type 2 immunity and basophil activation via IL-18.^28^

Our data suggest that the deficiency of mast cells, producers of histamine in the ECM, slightly impairs the disease pathology. More importantly, the abrogation of Tph1 and therefore of peripheral 5-HT leads to higher resistance and a significantly reduced mortality compared with the control group. Although we could not highlight any difference in terms of parasitemia or in the peripheral blood cell expansion, the abrogation of the synthesis of 5-HT leads to increased production of the alarmin IL-18, G-CSF, BAFF, VEGF-A, and IL-10 in Tph1-deficient mice. This cytokine signature in the blood is also correlated with a slightly significant increase in CD8^+^ T cells in peripheral lymph nodes. We and others have shown that macrocytosis occurs in the absence of 5-HT.^22^ Here, mice deficient in Tph1 show macrocytosis, more evident in the late phases of the infection, probably because of a reduced efficiency in erythropoiesis. Interestingly, Tph1 inhibitors reduce vascular remodeling and histamine release, protecting from pulmonary hypertension,^29^ which may explain the reason why the absence of Tph1 is indeed protective in mouse CM.

In a mouse model of CM, combining Tph1 inhibitors with antimalarial drugs controls both inflammation and parasitemia at the BBB, thereby preventing CM. We have additionally identified it as a potential pharmacological target for CM. Pairing this treatment with currently available antimalarial drugs improves malaria outcomes by preventing parasite dissemination to the brain.

Surprisingly, removing peripheral 5-HT synthesis significantly improves the clinical condition. The disease is less severe when Tph1 is absent, not because parasite growth is better controlled, as is evident from the level of systemic parasitemia in both strains of mice, but mainly because BBB permeability is reduced. Another important aspect to consider is that the absence of Tph1 leads to an increased production of tryptophan-derived metabolites, which are usually associated with microbiome amino acid metabolism.^30^^,^^31^^,^^32^ When Tph1 is absent, more tryptophan is available, which may lead to increased indole production.

In conclusion, the abrogation of peripheral 5-HT in the course of parasite infection may lead to significant control of BBB permeability, which then induces a profound regulation of the systemic inflammatory response against the parasite. This sort of vicious circle may represent a novel strategy to exploit to prevent CM disease.

### Limitations of the study

*cKit*^−/−^ (*KitW/KitW−v*) mice used in this study are often used to model mast-cell deficiency.^33^ However, the molecule cKIT is generally expressed in other cell types, such as melanocytes, duct cells, and innate lymphoid cells; they also lack intermediate cells derived from melanoblasts in the stria vascularis and exhibit macrocytic anemia.^34^^,^^35^^,^^36^ Therefore, we can’t exclude that their susceptibility to *Plasmodium* infection upon CM induction may also be due to the overall altered expression of cKIT on cell types other than mast cells in *cKit*^−/−^ mice. Thus, we have focused the manuscript on the specific role of Tph1.

In addition, a potential limitation of our study is that local parasite dissemination within the brain microvasculature was not directly quantified. However, our results strongly indicate that the striking protection observed in *Tph1*^−/−^ mice is mediated through the preservation of the host neurovascular unit rather than a reduction in parasite biomass. Indeed, despite displaying peripheral blood parasitemia kinetics identical to WT controls, *Tph1*^−/−^ mice were entirely protected from the clinical hallmarks of experimental CM. This was evidenced by a total absence of neurological symptoms and extensive maintenance of BBB integrity, as shown by the lack of Evans Blue extravasation (Figure 2C).

Another limitation of our tissue immunological analysis is that the data from the CLN (Figure 4B) represent relative frequencies rather than absolute cell numbers. However, this regional frequency signature must be read alongside the blood WBC, which revealed that infected *Tph1*^−/−^ mice harbor significantly fewer total peripheral blood WBCs compared with WT controls (Figure 3H). The lower systemic leukocyte burden in *Tph1*^−/−^ mice, paired with a distinct proportional priming profile in the lymph nodes, provides a mechanistic rationale for their complete clinical protection, as manifested by the reduced neurological scores and fully intact, Evans Blue-negative brains.

Finally, we could not measure serotonin upon treatment with telotristat. We recognize this as a limitation of our pharmacological validation. However, to relieve this limitation and robustly validate our findings, we utilized *Tph1*-deficient mice that genetically lack peripheral serotonin. Crucially, we observed that telotristat-treated WT mice closely mirrored the phenotype and disease outcomes of the untreated *Tph1*^−/−^ mice in our CM model. Furthermore, the potency of telotristat in rapidly reducing systemic 5-HT in mice is already established in the literature.^37^

## Resource availability

### Lead contact

Requests for further information and resources should be directed to and will be fulfilled by the lead contact, Teresa Zelante (teresa.zelante@unipg.it).

### Materials availability

All unique/stable reagents generated in this study are available from the lead contact with a completed materials transfer agreement.

### Data and code availability

• All data reported in this paper will be shared by the lead contact upon request.
• This paper does not report original code.
• Any additional information required to reanalyze the data reported in this paper is available from the lead contact upon request.

## Acknowledgments

The research was supported by the 10.13039/501100021856Italian Ministry of University and Research (MUR) National Innovation Ecosystem grant ECS00000041 – VITALITY.

## Author contributions

Methodology and investigation, editing, A.T., G. Rossini, L.V., G.V., M.M., C.F., A.S., V.O., G. Renga, A.M., M.T.A., A.B., C.C., N.G.; methodology, M.G., G.I., G.B., A.Y., F.F., and A.W.; conceptualization, writing, supervision, funding acquisition, T.Z. and R.S.

## Declaration of interests

The use of telotristat ethyl compound for the treatment of malaria, combination, and kit was registered and patented (WO2025078904A1).

## STAR★Methods

### Key resources table

**Table:** 

REAGENT or RESOURCE	SOURCE	IDENTIFIER
**Antibodies**		

Novocastra™ Liquid Mouse Monoclonal Anti-CD8 Antibody	Leica Biosystems	Cat# CD8-4B11-L-CE, RRID: AB_3107176
CD4	BD Biosciences	Cat# 553048; RRID AB_394585
CD8	BD Biosciences	AB_398527; RRID AB_398527

**Chemicals, peptides, and recombinant proteins**		

Telotristat ethyl	This paper	This paper
Evans blue dye	Catalog: E2129	Sigma-Aldrich
RPMI-1640 medium	Gibco	Cat# 11875093
DMSO	Sigma-Aldrich	Cat# 5.89569.0100
Penicillin-Streptomycin-Glutamine	Gibco	Cat# 10378-016
BSA	Miltenyi Biotec	Cat# 130-091-376

**Critical commercial assays**		

ProcartaPlex Mouse Immune Monitoring Panel 48-plex	Invitrogen	EPX480-20834-901; RRID:AB_3106560
CD8a^+^ T Cell Isolation Kit	Miltenyi Biotec	Order no. 130-104-075

**Experimental models: Organisms/strains**		

*Plasmodium berghei*	Malaria Research and Reference Reagent Resource Center (MR4), BEI Resources Repository, ATCC	Clone: MRA-311-ANKA (cl15cy1) BEI Resources; NIAID Cat# MRA-311, RRID:SCR_025406
*Anopheles stephensi*	Feldmann AM, Ponnudurai T et al., Med.Vet.Entomol^39^	RRID:SCR_025219
CD1, mouse	Charles River	Catalog: CRL:022 RRID:IMSR_CRL:022
C57BL/6 J, mouse strain	The Jackson Laboratory	Strain #:000664 RRID: IMSR_JAX:000664
*Tph1*^−/−^, mouse strain	Walther, D.J.et al., Cell^6^	N/A
*KitW/KitW−v*, mouse strain	The Jackson Laboratory	Strain #:100410 RRID:IMSR_JAX:100410

**Oligonucleotides**		

TPH1 GTCCCGGAAATCAAAGCAAAGA GGGCGAGTCCACCGAGAGG	Thermo Fisher	N/A

**Software and algorithms**		

GraphPad Prism 10.0	GraphPad Software	RRID:SCR_002798
QuPath version 0.3.1	Software Platform	RRID:SCR_018257
FACSDiva	BD Biosciences	RRID:SCR_001456
FlowJo version 10	Waters Biosciences	RRID:SCR_008520

### Experimental model and study participant details

#### Mouse model

Experiments with animals performed were carried out in accordance with the EU Animals Act 1986. Mosquito infections with *Plasmodium berghei* (*P. berghei*) by blood feeding on parasitised mice were approved by the Italian Ministry of Health and carried out under the License 1184/2020-PR based on the D.lgs. 26/2014. All the procedures were of mild-to-moderate severity, and protocols were designed to minimise the number of animals used. Opportunities for reduction, refinement, and replacement of animal procedures were constantly reviewed and considered.

Female and male C57BL/6J mice (6 to 8 weeks old) were purchased from Charles River for infection experiments. Mice were housed under specific pathogen-free conditions and kept on a 12-hour/12-hour day night cycle with access to water and standard rodent chow *ad libitum*. Female mice aged 8 to 10 weeks at the start of the experiment were used for all. For most experiments, at least 8-10 mice were used in each group, with multiple cages of mice (two to five mice per cage) used for each group to limit the possibility of cage effects. C57BL/6J (WT) and CD1 mice were obtained from Charles River Laboratory (Wilmington, Massachusetts, USA). *Tph1*^−/−^ mice were originally produced by M. Bader and D. Walther (Max Delbrück Center for Molecular Medicine, Berlin, Germany) in 2003 by classical embryonic stem cell technique on a Sv129 background, and animals were backcrossed for 10 generations to C57BL/6. The strain was donated to our institution and continuously kept as a homozygous breeding. Breeder pairs of *KitW/KitW−v* mice were purchased from Charles River Laboratory. For the *cKit* ^-/-^ mice, littermates were chosen for the experimental setting. For *Tph1*^-/-^ mice, control groups were maintained as separate homozygous breeding lines. However, to ensure rigour and reproducibility, all animals were matched for age, sex, and genetic background.

Mice were randomly chosen for the experimental groups. All mice were kept in the same mouse room under the same conditions, before and after infection with the parasite. For each experiment, at least 8-10 mice were used.

#### Mosquitoes

Mosquito strains were reared in a containment level 2 facility at the Department of Medicine and Surgery, Perugia, Italy, authorization N. PG/IC/Imp2/13/001-Rev2 from the Ministry of Health. *Anopheles stephensi* (Sind-Kasur Nijmegen strain) mosquitoes were raised at 27°C, 80% humidity, under a 12/12-hour light/dark cycle and maintained with 6% glucose containing the anti-fungal agent methylparaben (0.1%) (Sigma-Aldrich) under standard laboratory conditions. For infections, cryopreserved parasites were thawed and passed once *in vivo* before being used to infect experimental mice with 1 × 10^5^ diluted parasitized RBCs (200 μL) per mouse by intraperitoneal injection.

#### Mosquito infection with *Plasmodium berghei* parasites to obtain sporozoites

In brief, the parasite was routinely maintained by the alternating passage of blood-stage parasite (infected RBC) in CD1 mice (Charles River). *P.berghei* (ANKA strain) infected red blood cells 10^7^, were injected intraperitoneally into CD1 mice. Three days later, parasitemia was assessed by Giemsa staining (Hemacolor Rapid staining: Merck), and the presence of gametocytes (2–4%) was verified. *P. berghei ANKA-*infected mice were further adapted to nourish female *Anopheles stephensi* mosquitoes by infectious blood meal for the collection of sporozoites. Mosquitoes were deprived of glucose for 8 - 10 hours and then fed on anesthetized mice (1 mouse/cage/200 mosquitoes). Mice were anesthetized intraperitoneally with 100 mg/kg ketamine. The fed mosquitoes were then maintained on 10% sucrose soaked in cotton pads. The infection rate was checked on day 14 post blood feeding (PBF) in mosquitoes by microscopic examination (Evos FL Imaging System AMG) of the mosquito’s midgut for the availability of infectious oocysts. Sporozoites were collected on day 21 PBF from the mosquito’s salivary gland. Neubauer haemocytometry chamber was used for counting the number of sporozoites by optical microscopy, and 5 × 10^3^ sporozoites from the salivary gland of infected mosquitoes were injected in each experimental mouse intravenously.

### Method details

#### Mice infection, parasitemia levels and clinical conditions

The parasite was maintained by passage of infected RBC in C57BL/6J mice. For *in vivo* experiments, *P. berghei* (ANKA strain) infected red blood cells (10^5^), were injected intraperitoneally into C57BL/6J mice.

Parasitemia of mice was first evaluated 3 days post-infection, followed by daily measurement until the day of death. Two microliters of blood from infected mice were collected from the tail vein, and parasitemia was monitored by light microscopy on Giemsa-stained thin blood smears. More than 1000 erythrocytes were counted by microscopy to determine the percentage of parasitized cells. Animals were checked starting to 3 days daily for the development of neurological symptoms. A clinical score from 0 to 4 was employed as follows: 0 = normal, active; 1 = ruffled fur, less active; 2 = panting, coat staring; 3 = hunched, wobbly gait; 4 = convulsions and coma. Score 4 is a characteristic feature of ECM in susceptible mice infected with *P. berghei* ANKA.

#### Blood–brain barrier permeability assessment

The pathological vascular injury of the BBB was reflected by the leakage of plasma proteins across the BBB into the cerebral parenchyma. BBB permeability was assessed using the Evans blue assay. Infected WT, *cKit*^*−/−,*^ and *Tph1*^−/−^ mice were injected intravenously with 200 μL of 1% Evans blue dye in sterile physiological solution at day 10 post-infection. After two hours, mice were euthanized with the anesthetic (pentobarbital sodium), perfused intracardially with 10-20 mL physiological solution for the removal of intravascular-localized dye and brains collected. The brains were transferred to plastic plates and photographed. Then, the brains were weighed, mechanically dissociated and placed in Eppendorf tubes containing formamide at room temperature for 48 h to extract the Evans blue dye from the tissue. The optical density of the supernatant was measured at 620 nm (BMG LABTECH SPECTROstar nano spectrophotometer) and the amount of Evans blue staining in each group was calculated by a standard curve.

#### Histopathological analysis

Mice were euthanized by pentobarbital sodium on day 10; the brains and lymph nodes were collected immediately after euthanasia and were fixed in 10% neutral-buffered formalin, then they were embedded in paraffin. Examination of microvascular leakage and obstruction was made in 45-μm-thick sections stained with haematoxylin and eosin (H&E, kit ST Infinity H&E Staining System, Leica Biosystems) on automated Leica ST5020 Multistainer (Leica Biosystems, Nußloch). The slides obtained were scanned using an Aperio AT2 scanner (Leica Biosystems Nußloch) 20X objective lens. The signs of inflammation in all organs, in three non-contiguous sections per animal (*n* = 10/group), were visualized and analyzed using QuPath version 0.3.1 Software Platform; particularly, the number of macroscopic brain damage, such as hemorrhages and thrombi, was calculated as the means of counting 10 fields of each section.

#### Blood count

Blood was collected from mice at the indicated time points, drawn into heparinized tubes, and analyzed for total and differential blood parameter counts by using a laser hemocytometer (Sysmex XT 1800VET hematology analyzer, Sysmex).

#### Quantitative real-time polymerase chain reaction

Total RNA was extracted from targeted tissues using TRIzol Reagent (Thermo Fisher Scientific) following the manufacturer’s protocols and quantified by measuring the absorbance at 260 and 280 nm with TECAN spectrophotometer. RNA (1μg) per sample was reverse-transcribed to complementary DNA (cDNA) using the Luna Script RT Super Mix kit (Bio-Labs) and was stored at −80°C until use. RT involved a primer annealing at 25°C for 2 min, cDNA synthesis at 55°C for 10 min and Heat inactivation at 95°C for 1 min. Quantitative real-time PCR amplification and data analysis were performed on the Quant Studio 3 Applied Biosystem real-time system (Thermo Fisher Scientific) using Luna Universal qPCR Master (Bio-Labs, NE). PCR involved an initial denaturation at 95°C for 60 min, 45 cycles of 15 sec at 95°C and 30 sec at 60°C. Fluorescence readings were taken at 72°C after each cycle. At the end of each reaction, a melting curve (60 - 95°C) was checked to confirm the identity of the PCR product. Results are displayed as Fold change calculated by normalization to *Gapdh* mRNA. All measurements were performed in technical triplicate. Primers used for *Tph1* detec*tion:* GTCCCGGAAATCAAAGCAAAGA and GGGCGAGTCCACCGAGAGG.

#### Cytokine measurements

At day 10, when mice with ECM were terminally ill, they were killed and plasma collected for Luminex-based detection. Blood was taken by puncture of the heart at harvest with 25-gauge insulin syringes. All blood samples were collected by using heparin as an anticoagulant, they were centrifuged at 9000 rpm for 10 min, and supernatants were stored at −80°C until analyses. Luminex-based assays were designed to quantify the cytokine levels in total plasma.

#### Flow cytometry and cell magnetic separation

Flow cytometry was performed on a BD LSRFortessa™ cell analyser (Becton Dickinson) and the data were analyzed using FlowJo software (Tree Star). CD8^+^ T cells were isolated from the spleen of infected mice at day 10 post-inoculation of *Plasmodium berghei* sporozoites intravenously. Magnetic separation was based on the Miltenyi Biotech protocol for untouched CD8^+^ T cells.

#### Immunohistochemistry

4**-**μm-thick sections from previously formalin-fixed and paraffin-embedded (FFPE) tissue-blocks were obtained and subsequently stained with anti-CD8 (clone 4B11, ready-to-use—RTU—, Leica Biosystems, Newcastle Upon Tyne) and anti-TPH1 (polyclonal, dilution 1:200, RUO, St John Laboratory) antibodies respectively. The IHC were performed through the BOND III fully automated immunohistochemistry stainer (Leica Biosystems): in detail, for CD8, a heat-induced antigen retrieval (HIER) was used with the RTU EDTA based pH 9.0 Bond™ Epitope Retrieval Solution 2 (Leica Biosystems) for 20’, followed by primary antibody incubation for 15’. As regards TPH1, the HIER together with RTU citrate-based pH 6.0 Bond™ Epitope Retrieval Solution 1 (Leica Biosystems) for 30′ and a primary antibody incubation for 30′ were performed. Afterwards, the ready to use Bond™ Polymer Refine Detection System (Leica Biosystems) and the hematoxylin for nuclear counterstaining were used to visualize the immunoreaction.

CD8^+^ and Tph1^+^ T cells were identified and quantified as a percentage of positive cells in 10 consecutive 200X power fields, using QuPath version 0.3.1 Software Platform.

#### Metabolomic studies

The LC-MS based targeted metabolomic method was adapted from a previous developed protocol.^38^

All analyses were performed on an ACQUITY UPLC H-Class Bio system (Waters) coupled to a Xevo TQD triple quadrupole mass spectrometer equipped with an electrospray ionization source. Chromatographic separation employed an Acquity HSS T3 column (2.1 × 100 mm, 1.8 μm; Waters). Solvents of LC-MS grade were used throughout the study, including Milli-Q ultrapure water, methanol (Chebios), and formic acid (Sigma-Aldrich). Analytical standards for the investigated metabolites were obtained from Sigma-Aldrich.

The following compounds were included in the analysis:

• **Tryptophan pathway intermediates:** L-Tryptophan (Trp), L-Kynurenine (L-Kyn), Kynurenic acid (KYNA), Anthranilic acid (AA), 3-Hydroxykynurenine (3OH-Kyn), Xanthurenic acid (XA), 3-Hydroxyanthranilic acid (3OH-AA), Quinolinic acid (QA), Nicotinamide (NAM), 1-Methylnicotinamide (MNAM).
• **Serotonin/melatonin branch:** Serotonin (5HT), Melatonin (5-MT), 5-Hydroxy-L-tryptophan (5HTP), 5-Hydroxyindoleacetic acid (5-HIAA).
• **Indole derivatives:** Indole-3-acetic acid (I3AA), Indole-3-carboxaldehyde (I3A), Indole-3-propionic acid (IPA).
• **Phenylalanine/tyrosine metabolites:** L-Phenylalanine (Phe), Phenylpyruvic acid (PPA), L-Tyrosine (Tyr), 4-Hydroxyphenylpyruvic acid (4HPPA).

For accurate quantification, deuterated Quinolinic acid (4,5,6-D3) was used as the internal standard (QA-3D).

#### Sample preparation

Plasma aliquots were deproteinized by the addition of ice-cold methanol containing the internal standard. Following vortexing and sonication (30 min), samples were centrifuged at maximum speed for 10 min. The resulting supernatant was evaporated to dryness under a gentle nitrogen stream and reconstituted in 50 μL of 0.2% formic acid in water prior to LC-MS/MS injection.

#### Standard and calibration solutions

Primary stock solutions (100 mM) of all metabolites were prepared in methanol and stored at −80°C. Working solutions for calibration and quality controls were obtained by serial dilution. The calibration range was 0.0025–50 μM, with QC levels set at 0.025, 0.5, and 25 μM. Seven-point calibration curves were generated, and concentrations were determined using weighted linear regression (1/x^2^).

#### Chromatographic and mass spectrometry conditions

The chromatographic gradient was programmed from 0 to 90% methanol (mobile phase B, containing 0.2% formic acid) over 5 minutes, followed by a re-equilibration phase, with a flow rate of 0.4 mL/min. The injection volume was 5 μL, and the column temperature was maintained at 30°C.

The mass spectrometer was operated in positive electrospray ionization mode (ESI+), with source settings of: desolvation temperature 500°C, source temperature 150°C, desolvation gas flow 1000 L/h, and capillary voltage +2.7 kV. Detection was carried out in Multiple Reaction Monitoring (MRM) mode, applying optimized transitions for each analyte. Retention times and mass transitions were recorded to confirm metabolite identity and enable quantitative analysis.

#### 5-HT depletion with TPH1 inhibitor telotristat

Telotristat was dissolved in DMSO (2%) and diluted with 200μl PBS to obtain stock solutions for intraperitoneal injection of 50mg/Kg. In therapeutic experiments, these were started at day 3 after infection and administered on days 4 and 6. Control injections consisted of carrier solution only.

#### Synthesis of the TPH1 inhibitor telotristat

##### Step 1: Synthesis of 1-(2-bromo-4-chlorophenyl)-2,2,2-trifluoroethan-1-one (4)

**Figure:**
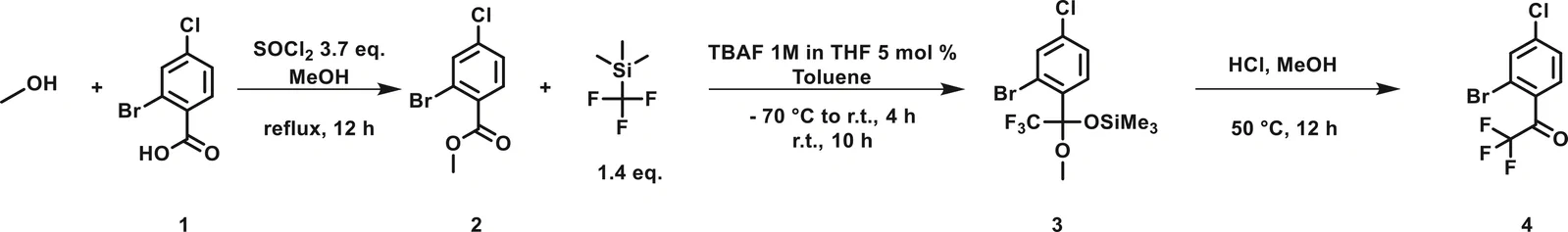

To a 100 mL RB flask equipped with a stirring bar under Argon inert atmospheren anhydrous MeOH (28 mL) was added and SOCl_2_ (37 mmol, 2.7 mL, 3.7 eq) was slowly dropped at 0°C. The ice water bath was removed and 2-bromo-4-chlorobenzoic acid **(1)** (10 mmol, 2.34 g) was added. The mixture was heated to reflux. After 12 h the reaction was stopped, the crude concentrated under reduced pressure and dissolved in DCM (15 mL). The organic phase was washed with water (10 mL), NaHCO_3_ satured solution (10 mL) and Brine (15 mL). The organic phase was dried with sodium sulphate and concentrated to give the 2-bromo-4-chloro-benzoic acid methyl ester **(2)** (9.2 mmol, 2.3 g, 92% yield), which was directly used in next step. 2-bromo-4-chloro-benzoic acid methyl ester **(2)** (9.2 mmol, 2.3 g) in anhydrous toluene (36 mL) was cooled down to – 70°C under argon inert atmosphere and trifluoromethyl trimethyl silane (12.9 mmol, 1.83 g, 1.99 mL) (1.4 eq.) was added. TBAF 1 M in THF (5 mol %,0.46 mmol, 0.46 mL) was added dropwise, and the mixture allowed to warm up to room temperature over 4 h after which it was stirred for 10 hours at room temperature. The reaaction was stopped and the mixture concentrated. The crude intermediate **(3)** was dissolved in MeOH 17 mL and a solution of HCl 6 N (17 mL) was dropped at 0°C. The mixture was left under stirring at 50°C. After 12 h the reaction was stopped and MeOH was removed and the crude extracted with DCM (37 mL). The organic phase was washed with water (15 mL), NaHCO_3_ (15 mL), brine (15 mL), dried over sodium sulphate and concentrated under reduced pressure. The crude was purified by chromatohgraphic column PE/EtOAc (EtOAc from 1 % to 5 %) to obtain the desired product 1-(2-bromo-4-chlorophenyl)-2,2,2-trifluoroethan-1-one **(4)** (6.27 mmol, 1.8 g, 63 % isolated yield).

##### Step 2: Synthesis of (*R*)-1-(2-bromo-4-chlorophenyl)-2,2,2-trifluoroethan-1-ol (5)

**Figure:**
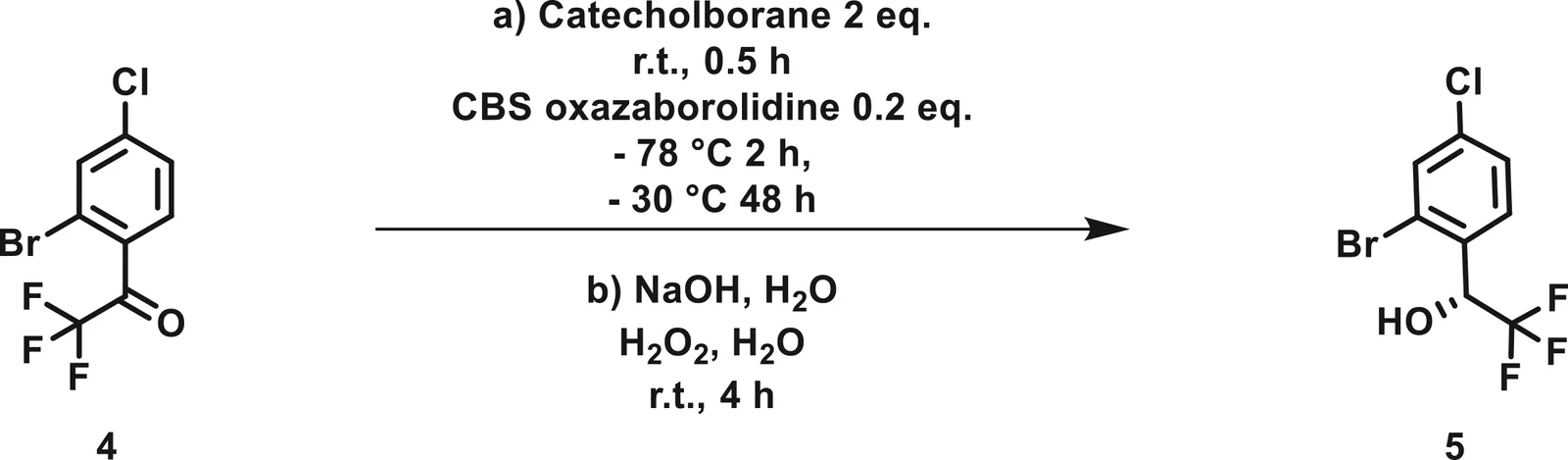

In a 50 mL RB flask equipped with a stirring bar Catecholborane 1M in THF (11. 9 mmol, 11.9 mL) (2 eq) and S-2-methyl-CBS oxazaborolidine (1.19 mmol, 329 mg) (0.2 eq) were consecutively added under Argon inert atmosphere and the resulting mixture was left under stirring at room temperature. After 0.5 h the reaction was cooled down to – 78°C and 1-(2-bromo-4-chloro-phenyl)-2,2,2-trifluoroethan-1-one **(4)** (5.95 mmol, 1.7 g) in anhydrous THF (17 mL) was added dropwise over 2 hours. The reaction mixture was allowed to warm up to – 30°C and stirred for 48 hours. NaOH 3 N solution (10 mL) was added and the cooling bath was replaced by an ice-water bath. Then a 30% hydrogen peroxide solution in water (10 mL) was added dropwise over 15 min. The mixture was stirred at room temperature. After 4 hours the reaction was stopped and the mixture was extracted with DEE (4 x 15 mL). The combined organic phases were washed with NaOH 1 N solution (4 x 15 mL) and Brine (2 x 15 mL), dried with sodium sulphate, filtered and concentrated under reduced pressure. The crude was purified by chromatographic column PE/EtOAc (EtOAc from 2% to 10%) to obtain the desired product (*R*)-1-(2-bromo-4-chlorophenyl)-2,2,2-trifluoroethan-1-ol **(5)** (4.42 mmol, 1.28 g, 74% isolated yield).

##### Step 3: Synthesis of (*R*)-1-(4-chloro-2-(3-methyl-1*H*-pyrazol-1-yl)phenyl)-2,2,2-trifluoroethan-1-ol (7)

**Figure:**
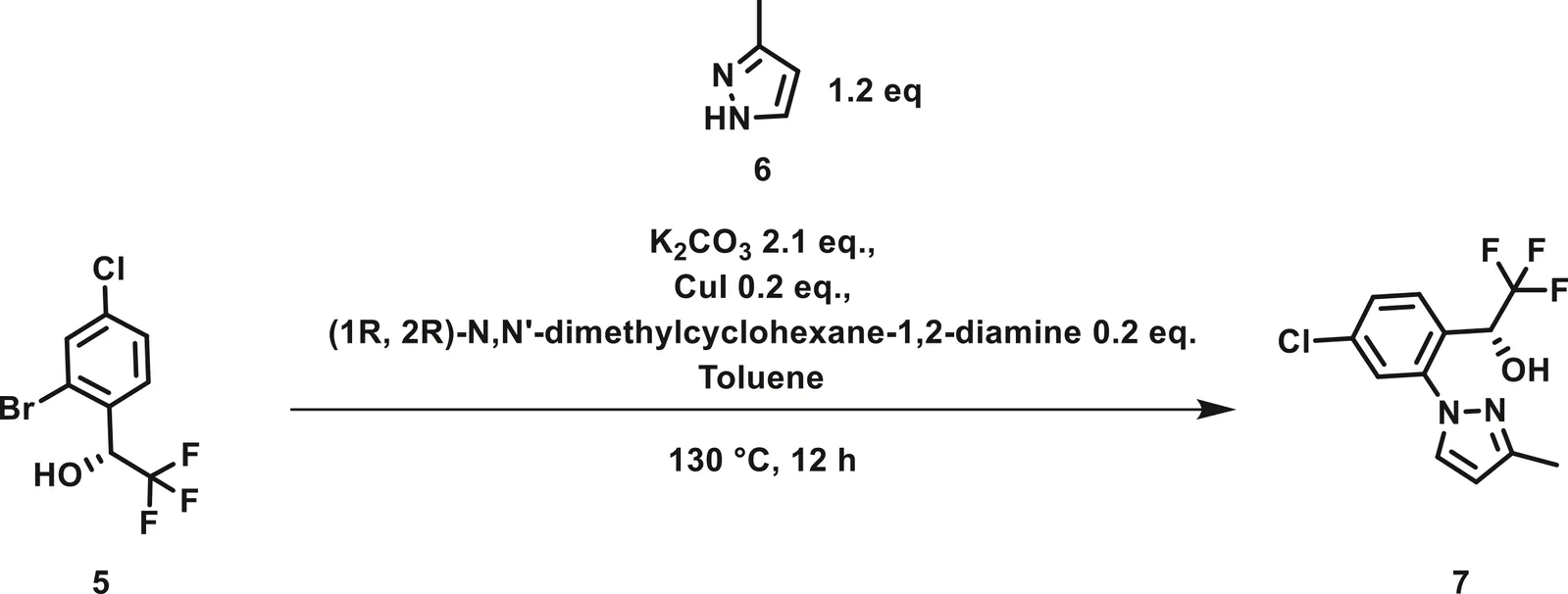

In a 12 mL pressure tube equipped with a stirring bar (*R*)-1-(2-bromo-4-chlorophenyl)-2,2,2-trifluoroethan-1-ol **(5)** (3.45 mmol, 1 g), 3-methylpyrazole **(6)** (4.14 mmol, 339.93 mg, 0.320 mL), (1.2 eq), CuI (0.69 mmol, 131.41 mg) (0.2 eq), potassium carbonate (7.2 mmol, 995 mg) (2.1 eq), (1R, 2R)-N,N′-dimethylcyclohexane-1,2-diamine (0.69 mmol, 98.14 mg) (0.2 eq) and Toluene 5.07 mL were consecutively added. The tube was sealed and the mixture was heated at 130°C and left under stirring. After 12 h the reaction was stopped and cooled to room temperature. EtOAc (30 mL) was added. The organic phase was washed with water (4 x 15 mL) and Brine (15 mL), dried with sodium sulphate, filtered and concentrated. The crude was purified by chromatographic column PE/EtOAc (EtOAc from 0 to 20%) to obtain the desired product (*R*)-1-(4-chloro-2-(3-methyl-1*H*-pyrazol-1-yl)phenyl)-2,2,2-trifluoroethan-1-ol **(7)** (2.36 mmol, 686 mg, 69% isolated yield).

##### Synthesis of ethyl (*S*)-3-(4-(2-amino-6-chloropyrimidin-4-yl)phenyl)-2-((*tert*-butoxycarbonyl)amino)propanoate (8)

**Figure:**
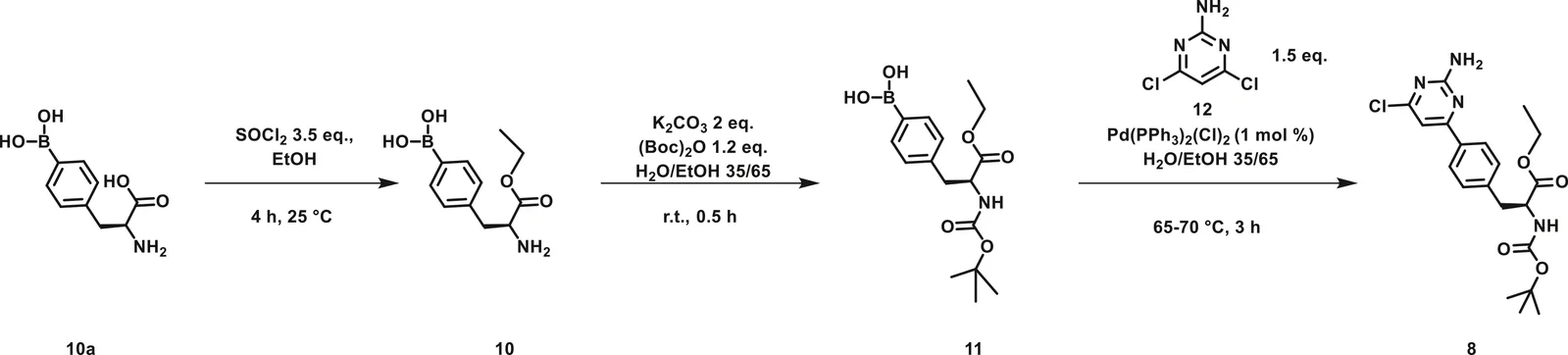

In a screw capped vial anhydrous EtOH (1.57 mL), SOCl_2_ (5.04 mmol, 600 mg, 0.366 mL) (3.5 eq) and (S)-2-amino-3-(4-boronophenyl)propanoic acid **(10a)** (1.44 mmol, 300 mg) were consecutively added at 0 °C, the vial was sealed and the mixture was left under stirring at 25°C. After 4 h water (2.5-3 mL) was slowly added at 0°C, then EtOH (4.32 mL), potassium carbonate (2.88 mmol, 398 mg) (2 eq) and (Boc)_2_O (1.73 mmol, 377 mg, 0.369 mL) (1.2 eq) were consecutively added and the mixture was left under stirring at room temperature. After 2 h 4,6-dichloropyrimidin-2-amine **(12)** (2.16 mmol, 354 mg) (1.5 eq) and Pd(PPh_3_)Cl_2_ (0.0144 mmol, 10.08 mg) (1 mol %) were consecutively added and the mixture was left under stirring at 70°C. After 3 h the reaction was stopped, the reaction medium was evaporated, and water (30 mL) and EtOAc (20 mL) were added (some drops of HCl 1 N were added to the aqueous phase to neutralize the mixure). The aqueous phase was extracted with EtOAc (3 x 20 mL) and the organic phase is washed with Brine (20 mL), dried with sodium sulphate, filtered and concentrated under reduced pressure. The crude was purified by chromatographic column PE/EtOAc (EtOAc from 0 to 30%) to obtain the desired product ethyl (*S*)-3-(4-(2-amino-6-chloropyrimidin-4-yl)phenyl)-2-((*tert-*butoxycarbonyl)amino)propanoate **(8)** (0.42 mmol, 177 mg, 29% isolated yield).

##### Step 4: Synthesis of (*S*)-3-(4-(2-amino-6-((*R*)-1-(4-chloro-2-(3-methyl-1H-pyrazol-1-yl)phenyl)-2,2,2-trifluoroethoxy)pyrimidin-4-yl)phenyl)-2-((*tert*-butoxycarbonyl)amino)propanoic acid (9)

**Figure:**
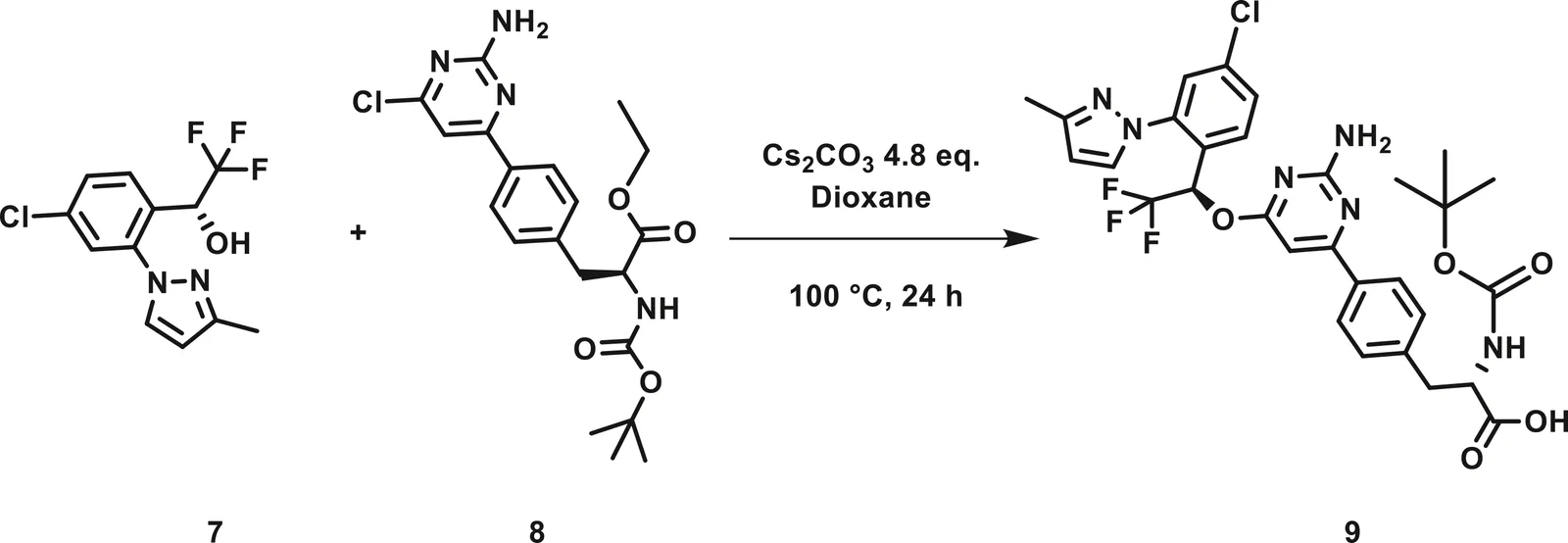

In a screw capped vial compound (*R*)-1-(4-chloro-2-(3-methyl-1*H*-pyrazol-1-yl)phenyl)-2,2,2-trifluoroethan-1-ol **(7)** (0.85 mmol, 247 mg) (1.2 eq), compound ethyl (*S*)-3-(4-(2-amino-6-chloropyrimidin-4-yl)phenyl)-2-((*tert*-butoxycarbonyl)amino)propanoate **(8)** (0.71 mmol, 300 mg), dioxane (3.48 mL) and Cs_2_CO_3_ (3.45 mmol, 1.11 g) (4.8 eq) were consecutively added, the vial was sealed and the reaction was left under stirring at 100°C. The reaction was stopped after 24 h. To the reaction mixture was added EtOAc (20 mL) and water (20 mL) and the mixture was transferred in a separating funnel. The aqueous phase was washed with EtOAc (3 x 20 mL), DEE (20 mL), CHCl_3_ (5 mL), THF (5 mL) and DCM (5 mL) to completely extract the product. The combined organic phases were dried with sodium sulphate, filtered and concentrated to obtain the crude. The crude was purified by chromatographic column DCM/EtOH (EtOH from 5 to 30%) to obtain the desired product (*S*)-3-(4-(2-amino-6-((*R*)-1-(4-chloro-2-(3-methyl-1*H*-pyrazol-1-yl)phenyl)-2,2,2-trifluoroethoxy)pyrimidin-4-yl)phenyl)-2-((*tert*-butoxycarbonyl)amino)propanoic acid **(9)** (0.32 mmol, 208 mg, 45% isolated yield).

##### Step 5: ethyl (S)-2-amino-3-(4-(2-amino-6-((R)-1-(4-chloro-2-(3-methyl-1H-pyrazol-1-yl)phenyl)-2,2,2-trifluoroethoxy)pyrimidin-4-yl)phenyl)propanoate (Xermelo or Telotristat ethyl)

**Figure:**
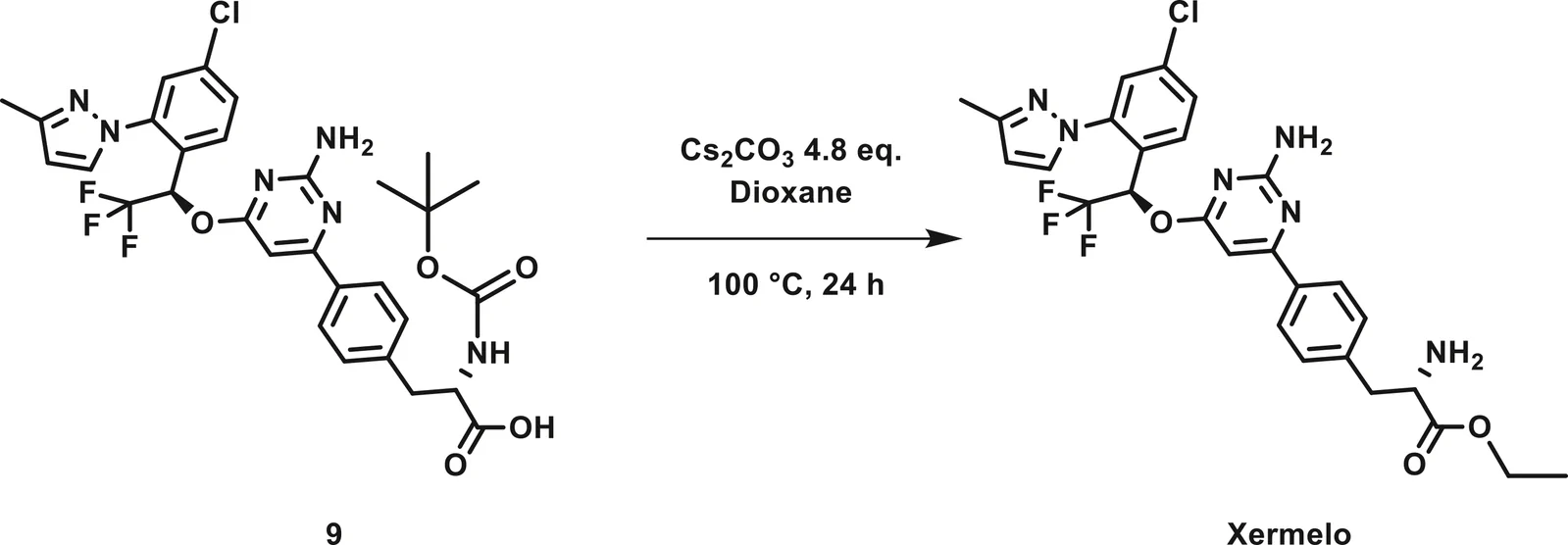

In a screw capped vial anhydrous EtOH (3 mL) was added. SOCl_2_ (2.17 mmol, 258 mg, 160 μL) (7 eq) was added dropwise at 0°C under stirring. After 10 min compound (*S*)-3-(4-(2-amino-6-((*R*)-1-(4-chloro-2-(3-methyl-1H-pyrazol-1-yl)phenyl)-2,2,2-trifluoroethoxy)pyrimidin-4-yl)phenyl)-2-((*tert*-butoxycarbonyl)amino)propanoic acid **(9)** (0.31 mmol, 200 mg) was added at 0°C under stirring. The temperature was increased to 45°C and the mixture left under stirring. After 12 h the reaction was stopped and Na_2_CO_3_ satured solution was added at 0°C under stirring (up to pH 10). The aqueous phase was extracted with EtOAc (5 x 5 mL). The combined organic phases were dried with sodium sulphate, filtered and concentrated under reduced pressure to obtain the crude. The crude was purified by chromatographic column (DCM/EtOH, EtOH from 2% to 4%) to obtain the desired final product ethyl (*S*)-2-amino-3-(4-(2-amino-6-((*R*)-1-(4-chloro-2-(3-methyl-1*H*-pyrazol-1-yl)phenyl)-2,2,2-trifluoroethoxy)pyrimidin-4-yl)phenyl)propanoate **Xermelo**. (0.19 mmol, 110 mg, 61% isolated yield).

#### Quantitative real-time polymerase chain reaction

Total RNA was extracted from targeted tissues using TRIzol Reagent (Thermo Fisher Scientific) following the manufacturer’s protocols and quantified by measuring the absorbance at 260 and 280 nm with TECAN spectrophotometer. RNA (1μg) per sample was reverse-transcribed to complementary DNA (cDNA) using the Luna Script RT Super Mix kit (Bio-Labs) and was stored at −80°C until use. RT involved a primer annealing at 25°C for 2 min, cDNA synthesis at 55°C for 10 min and Heat inactivation at 95°C for 1 min. Quantitative real-time PCR amplification and data analysis were performed on the Quant Studio 3 Applied Biosystem real-time system (Thermo Fisher Scientific) using Luna Universal qPCR Master (Bio-Labs, NE). PCR involved an initial denaturation at 95°C for 60 min, 45 cycles of 15 sec at 95°C and 30 sec at 60°C. Fluorescence readings were taken at 72°C after each cycle. At the end of each reaction, a melting curve (60 - 95°C) was checked to confirm the identity of the PCR product. Results are displayed as Fold change calculated by normalization to *Gapdh* mRNA. All measurements were performed in technical triplicate. Primers used for *Tph1* detec*tion:* GTCCCGGAAATCAAAGCAAAGA and GGGCGAGTCCACCGAGAGG.

#### Cytokine measurements

At day 10, when mice with ECM were terminally ill, they were killed and plasma collected for Luminex-based detection. Blood was taken by puncture of the heart at harvest with 25-gauge insulin syringes. All blood samples were collected by using heparin as an anticoagulant, they were centrifuged at 9000 rpm for 10 min, and supernatants were stored at −80°C until analyses. Luminex-based assays were designed to quantify the cytokine levels in total plasma.

#### Flow cytometry

Flow cytometry was performed on a BD LSRFortessa™ cell analyzer (Becton Dickinson) and the data were analyzed using FlowJo software (Tree Star).

#### Immunohistochemistry

4**-**μm-thick sections from previously formalin-fixed and paraffin-embedded (FFPE) tissue-blocks were obtained and subsequently stained with anti-CD8 (clone 4B11, ready-to-use—RTU—, Leica Biosystems, Newcastle Upon Tyne) and anti-TPH1 (polyclonal, dilution 1:200, RUO, St John Laboratory) antibodies respectively. The IHC were performed through the BOND III fully automated immunohistochemistry stainer (Leica Biosystems): in detail, for CD8, a heat-induced antigen retrieval (HIER) was used with the RTU EDTA based pH 9.0 Bond™ Epitope Retrieval Solution 2 (Leica Biosystems) for 20’, followed by primary antibody incubation for 15’. As regards TPH1, the HIER together with RTU citrate-based pH 6.0 Bond™ Epitope Retrieval Solution 1 (Leica Biosystems) for 30’ and a primary antibody incubation for 30’ were performed. Afterwards, the ready to use Bond™ Polymer Refine Detection System (Leica Biosystems) and the hematoxylin for nuclear counterstaining were used to visualize the immunoreaction.

CD8^+^ and Tph1^+^ T cells were identified and quantified as a percentage of positive cells in 10 consecutive 200X power fields, using QuPath version 0.3.1 Software Platform.

### Quantification and statistical analysis

Statistical analyses were performed using GraphPad Prism 10.0.3 (GraphPad Software version). Survival data were analysed by the log-rank (Mantel–Cox) test. Significance of data from experimental groups compared with control groups was determined by unpaired *t*-test with or without Welch’s correction, Mann-Whitney *U*-test, one-way analysis of variance (ANOVA) with Bonferroni’s multiple-comparison test, two-way ANOVA test with Bonferroni’s or Tukey’s multiple-comparison tests, or the Kruskal-Wallis *H*-test with Dunn’s multiple-comparisons test. Values of *p* ≤ 0.05 were considered statistically significant, and all data are displayed as mean ± SEM of results from a single representative experiment performed in duplicate and/or in triplicate. ∗*p* < 0.05, ∗∗*p* < 0.01, ∗∗∗*p* < 0.001, ∗∗∗∗*p* < 0.0001. Each experiment was repeated at least twice to confirm reproducibility. Statistical details can be found in the figure legends.

## Supplemental information

Document S1. Figures S1–S5

Video abstract. Deficiency of peripheral serotonin caused by Tph1 abrogation attenuates cerebral malaria severity
