# Supplementary material for: Deficiency of peripheral serotonin caused by Tph1 abrogation attenuates cerebral malaria severity
Source: iScience. 2026 Oct 29;29(11):118002. doi: 10.1016/j.isci.2026.118002 (PMC13739000; doi:10.1016/j.isci.2026.118002)
Supplement: Supplementary file 1 [file mmc1.pdf]

iScience, Volume *29*

Supplemental information

Deficiency of peripheral serotonin caused by Tph1 abrogation attenuates cerebral malaria severity

Antonella Turco, Gabriele Rossini, Luigi Vaccaro, Gianluca Vascelli, Martina Mandarano, Claudia Floridi, Angelo Sidoni, Giorgia Renga, Arianna Miglio, Maria Teresa Antognoni, Aurora Barbetta, Carolina Colliva, Nicola Giacchè, Marco Gargaro, Alessia Sulla, Gloria Iacomelli, Max Lombardi, Giulia Bicchieraro, Alicia Y.W. Wong, Francesca Fallarino, Vasileios Oikonomou, Teresa Zelante, and Roberta Spaccapelo

SUPPLEMENTAL FIGURES

PARAMETER	STATUS	SCORE
**Appearance**		
	Normal	0
	Coat ruffled	1
	Coat starling; panting	2
	Dead	3
**Natural behavior**		
	Normal	0
	Hunched/wobbly gait	1
	Partial paralysis/immobile	2
	Convulsions/coma	3
	Dead	4

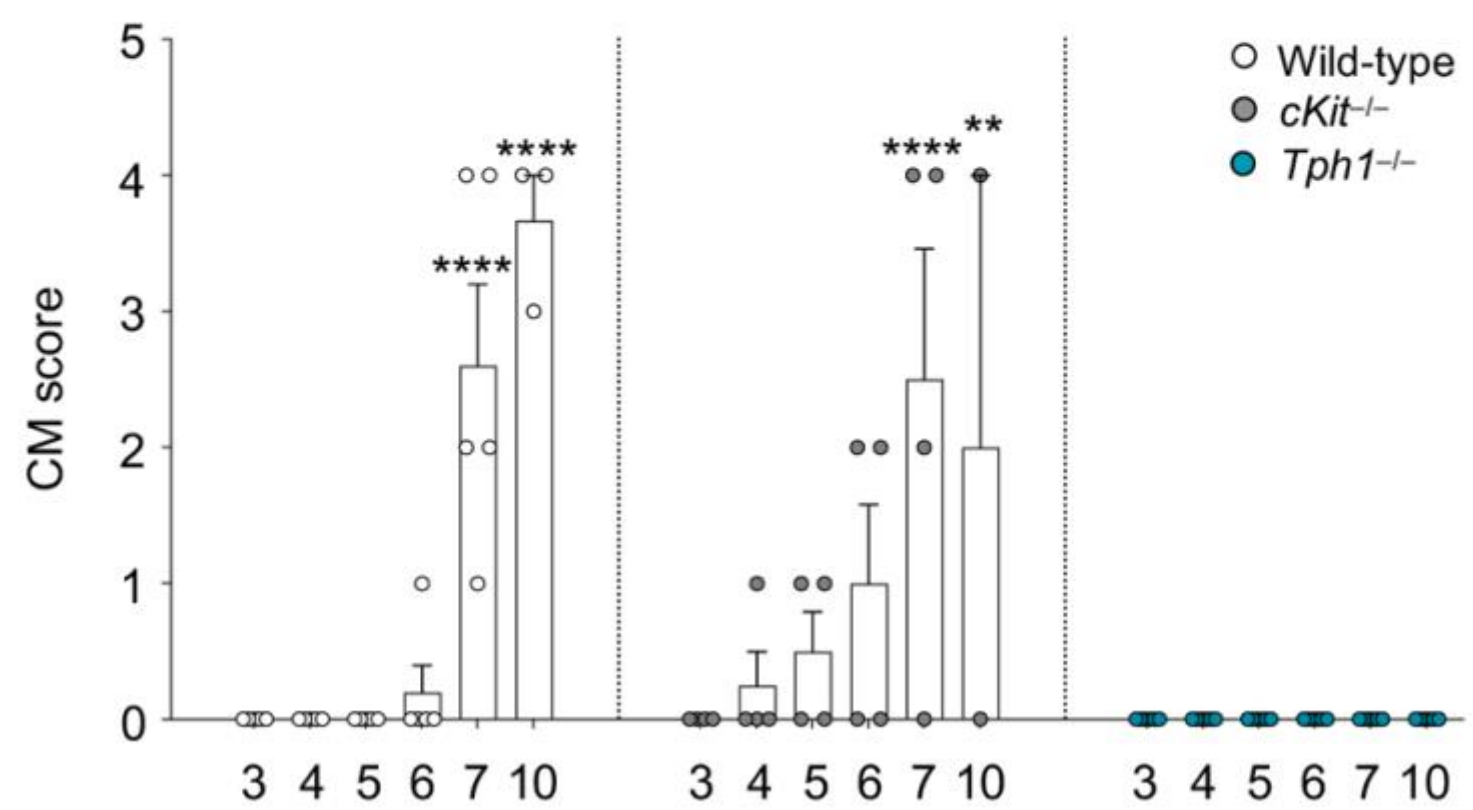

Figure S1, related to Figure 1 and Figure 2. Peripheral 5-HT–deficient mice do not show neurological symptoms during *Plasmodium berghei* ANKA infection. Table of pathological parameters with corresponding scores (top panel) and cerebral malaria (CM) score of wild-type, *cKit*$^{-/-}$, and *Tph1*$^{-/-}$ mice **infected with *Plasmodium berghei* sporozoites intravenously** (bottom panel) (*n* = 4 per group; three experiments). Each dot represents an individual mouse. All quantitative data represent mean ± SEM. ***P* < 0.01, *****P* < 0.0001, as calculated using two-way ANOVA with Dunn's multiple-comparison test.

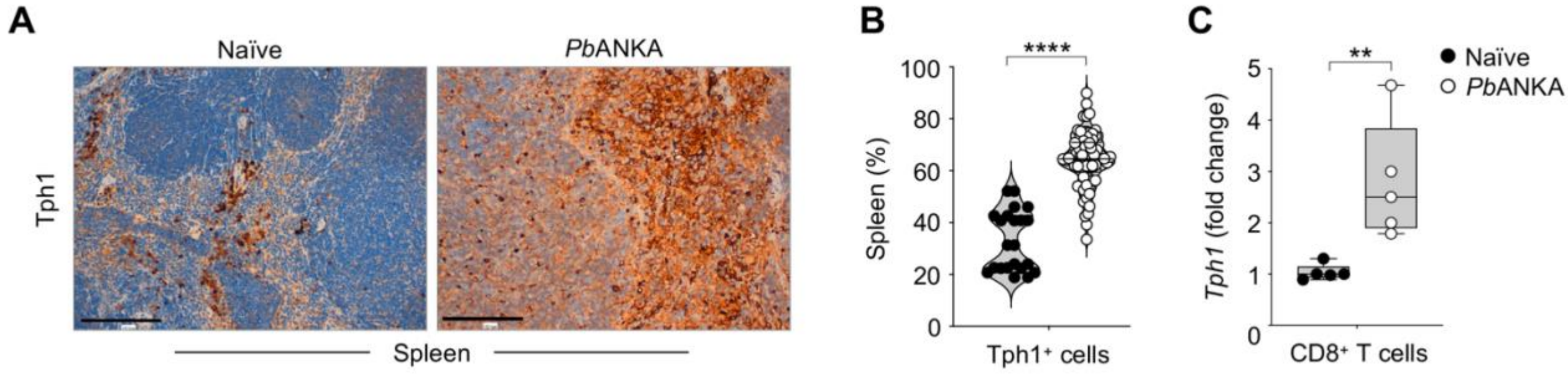

Figure S2, related to Figure 4. Both Tph1^{+} and Tph1^{+} CD8^{+} T cells were recruited in the spleen of mice infected with *Plasmodium berghei* sporozoites intravenously.

(**A** and **B**) Representative immunohistochemical images (A) and percentage (B) of Tph1^{+} T cells in spleen tissues from naïve and *PbA*-infected mice (n = 22 to 70 per group; four experiments). (C) Relative fold change of *Tph1* gene in CD8^{+} T cells (n = 5 per group; two experiments). Each dot represents an individual mouse. **P < 0.01, ****P < 0.0001 as calculated using Mann-Whitney U-test (B and C).

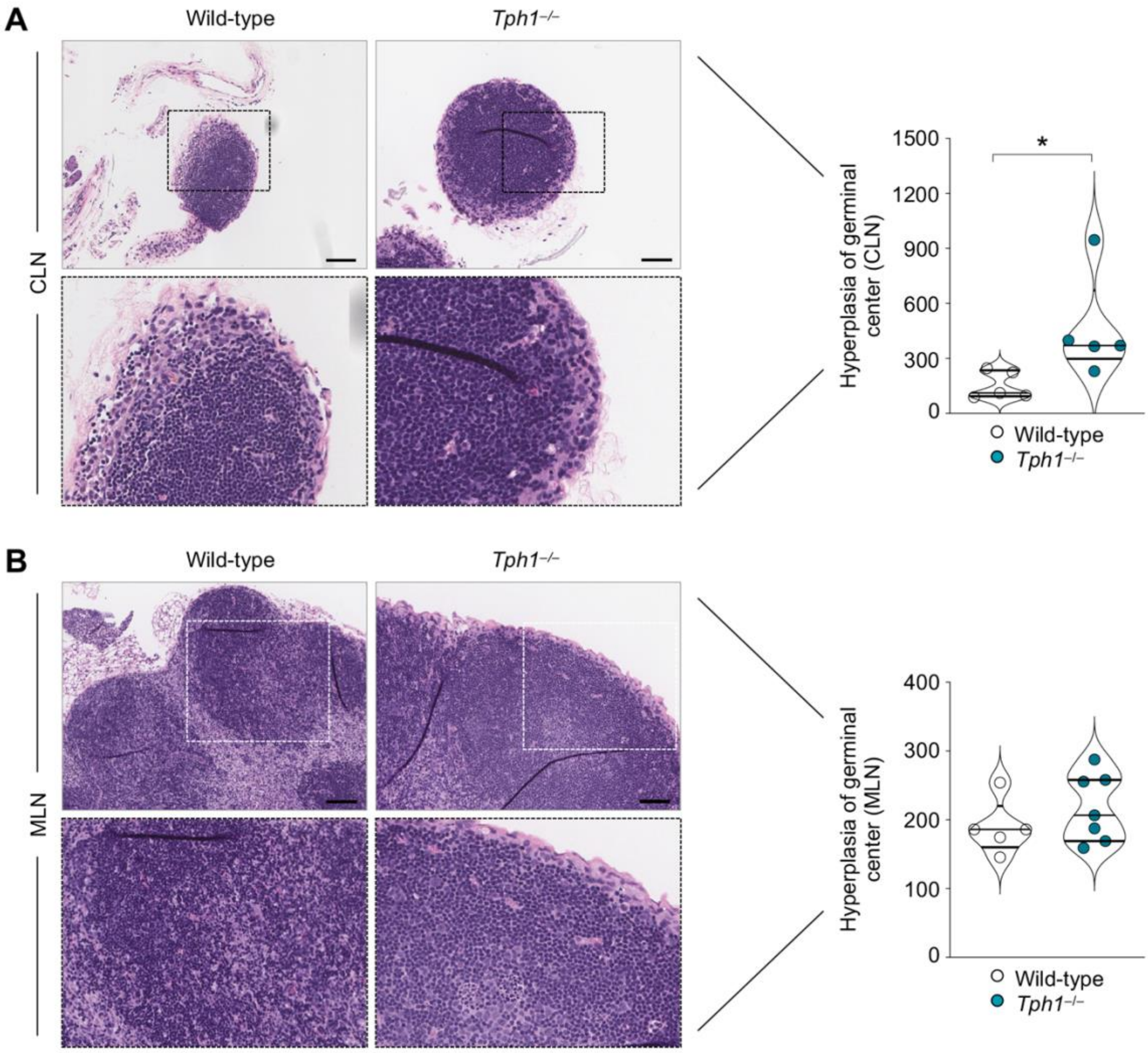

Figure S3, related to Figure 4. 5-HT peripheral-deficient mice displayed normal development of germinal centres in peripheral lymph nodes. (**A**) Representative images of H&E-stained cervical lymph node (CLN) sections (top left panels) and quantification of germinal center hyperplasia (top right panel) in wild-type and *Tph1*$^{-/-}$ mice infected as described above (*n* = 5 per group; two experiments). (**B**) Representative images of H&E-stained mesenteric lymph node (MLN) sections (bottom left panels) and quantification of germinal center hyperplasia (bottom right panel) in wild-type and *Tph1*$^{-/-}$ mice infected as described above in Figure S1 (*n* = 5 to 7 per group; two experiments). Each dot represents an individual mouse. All quantitative data represent mean ± SEM. *$P < 0.05$ as calculated using Mann-Whitney *U*-test (A).

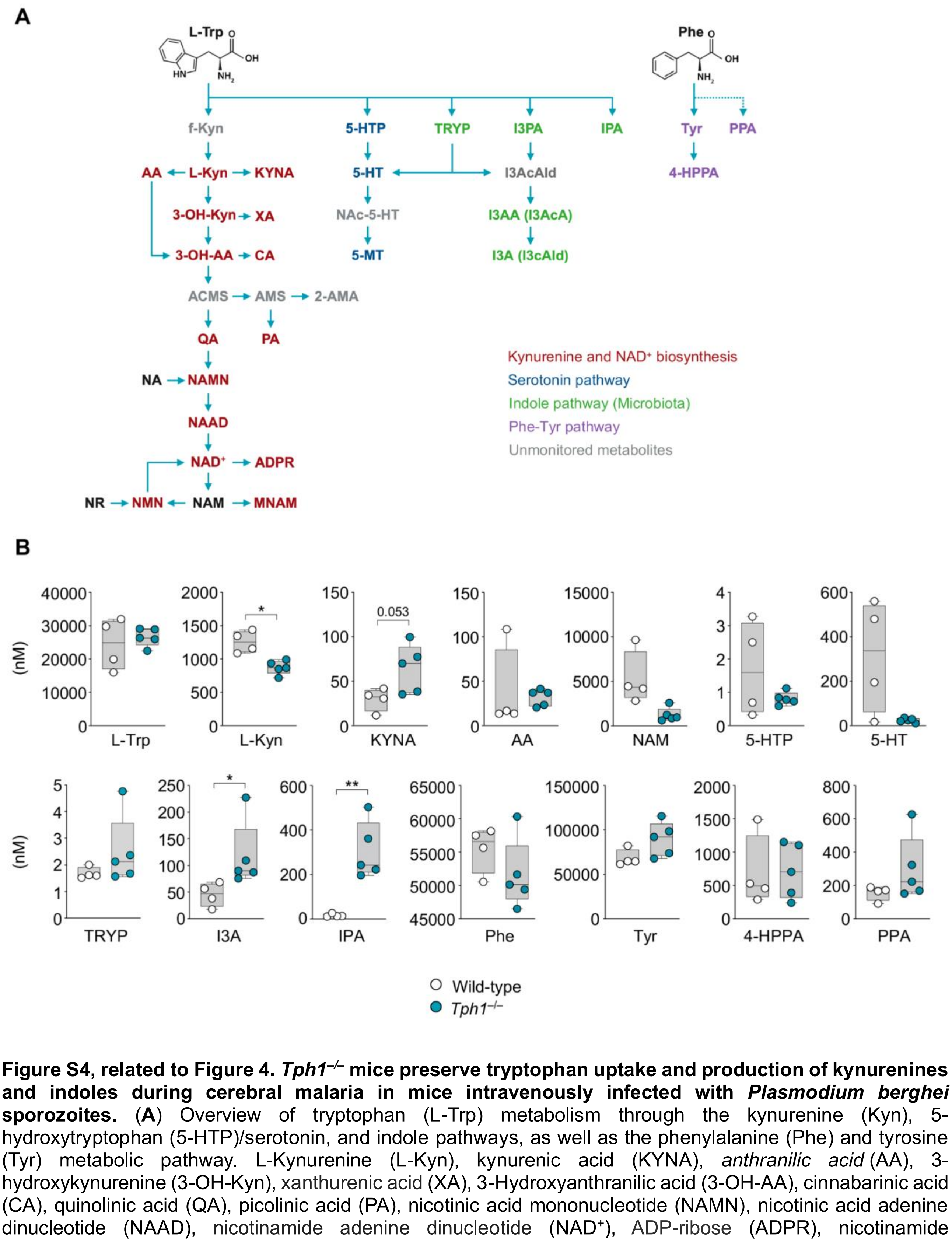

Figure S4, related to Figure 4. *Tph1*$^{-/-}$ mice preserve tryptophan uptake and production of kynurenines and indoles during cerebral malaria in mice intravenously infected with *Plasmodium berghei* sporozoites. (**A**) Overview of tryptophan (L-Trp) metabolism through the kynurenine (Kyn), 5-hydroxytryptophan (5-HTP)/serotonin, and indole pathways, as well as the phenylalanine (Phe) and tyrosine (Tyr) metabolic pathway. L-Kynurenine (L-Kyn), kynurenic acid (KYNA), *anthranilic acid* (AA), 3-hydroxykynurenine (3-OH-Kyn), xanthurenic acid (XA), 3-Hydroxyanthranilic acid (3-OH-AA), cinnabarinic acid (CA), quinolinic acid (QA), picolinic acid (PA), nicotinic acid mononucleotide (NAMN), nicotinic acid adenine dinucleotide (NAAD), nicotinamide adenine dinucleotide (NAD$^+$), ADP-ribose (ADPR), nicotinamide mononucleotide (NMN), *N1-methylnicotinamide* (MNAM), 5-Hydroxytryptophan (5-HTP), *serotonin* (5-HT), 5-methyl tryptophan (5-MT), tryptamine (TRYP), indole-3-acetamide (I3PA), indole-3-acetic acid (I3AA), indole-3-aldehyde (I3A), indole-3-propionic acid (IPA), phenylpyruvic acid (PPA) and 4-hydroxyphenylpyruvic acid (4-HPPA). (**B**) Plasma metabolite production in *PbA*-infected wild-type and *Tph1*$^{-/-}$ mice (n = 4 per group; two experiments). Each dot represents an individual mouse. All quantitative data represent mean ± SEM. *P < 0.05, **P < 0.01, ****P < 0.0001 as calculated using unpaired t-test, or unpaired t-test with Welch's correction (B), 5-HTP.

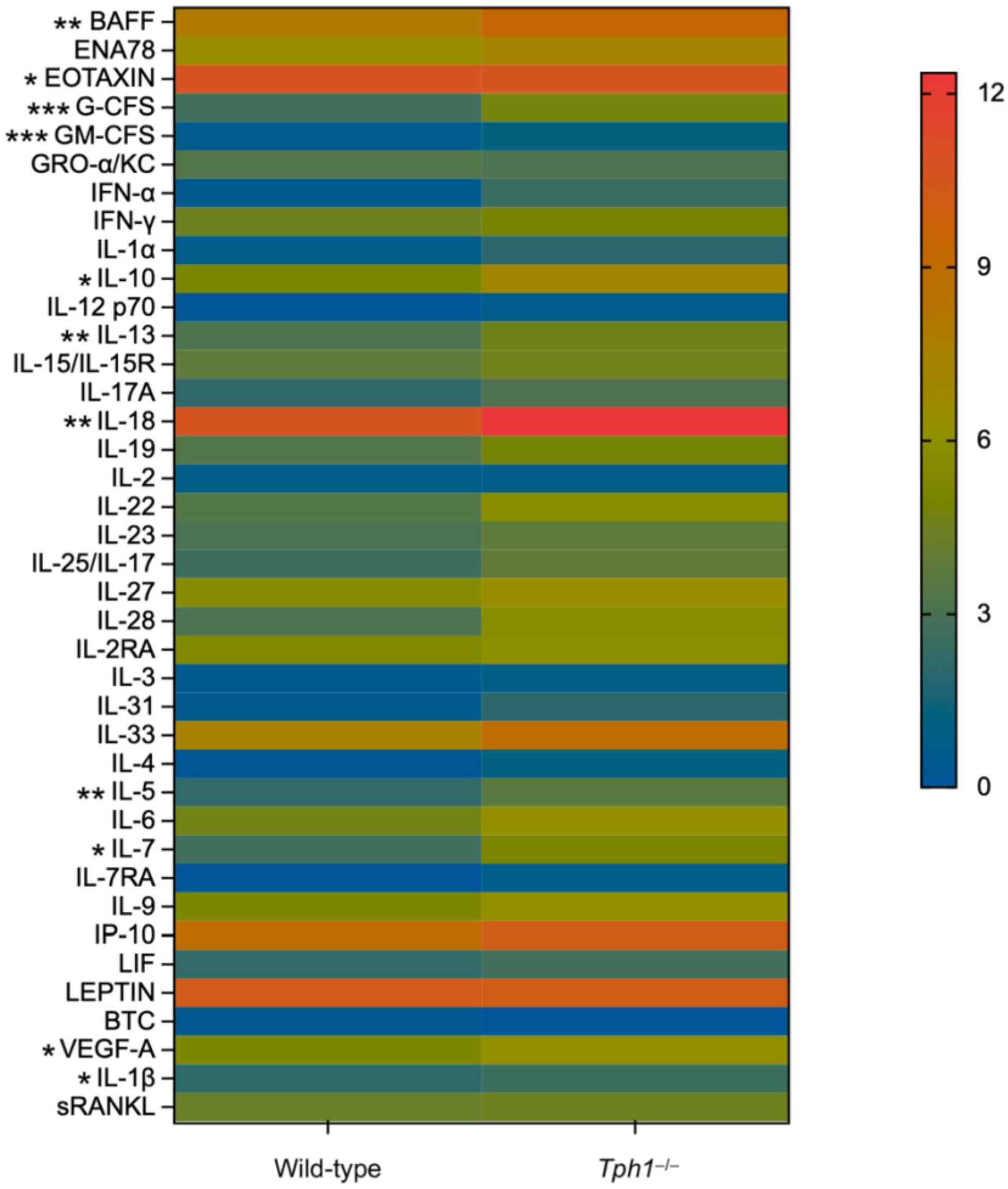

Figure S5, related to Figure 4. Heatmap of cytokine concentrations in wild-type and *Tph1*$^{-/-}$ mice following intravenous injection of *Pb*A sporozoites. Data are shown as $\log_2$(pg/mL + 1). Cytokines marked with asterisks were significantly different between groups. Corresponding quantitative data for BAFF, EOTAXIN, G-CSF, IL-18, and VEGF-A are shown in Fig. 4A.
